# Recurrent planetesimal formation in an outer part of the early solar system

**DOI:** 10.1038/s41598-024-63768-4

**Published:** 2024-07-01

**Authors:** Wladimir Neumann, Ning Ma, Audrey Bouvier, Mario Trieloff

**Affiliations:** 1 Institute of Geodesy and Geoinformation Science, Technische Universität Berlin, Kaiserin-Augusta-Allee 104-106, 10553 Berlin, Germany; 2 Klaus-Tschira-Labor für Kosmochemie, Institut für Geowissenschaften, Universität Heidelberg, Im Neuenheimer Feld 234-236, 69120 Heidelberg, Germany; 3 Institute of Planetary Research, German Aerospace Center (DLR), Rutherfordstr. 2, 12489 Berlin, Germany; 4 Institute of Geochemistry and Petrology, ETH Zürich, Sonneggstrasse 5, 8092 Zurich, Switzerland; 5 Bayerisches Geoinstitut, University of Bayreuth, 95447 Bayreuth, Germany

**Keywords:** Asteroids, comets and Kuiper belt, Early solar system

## Abstract

The formation of planets in our solar system encompassed various stages of accretion of planetesimals that formed in the protoplanetary disk within the first few million years at different distances to the sun. Their chemical diversity is reflected by compositionally variable meteorite groups from different parent bodies. There is general consensus that their formation location is roughly constrained by a dichotomy of nucleosynthetic isotope anomalies, relating carbonaceous (C) meteorite parent bodies to the outer protoplanetary disk and the non-carbonaceous (NC) parent bodies to an origin closer to the sun. It is a common idea, that in these inner parts of the protoplanetary disks, planetesimal accretion processes were faster. Testing such scenarios requires constraining formation ages of meteorite parent bodies. Although isotopic age dating can yield precise formation ages of individual mineral constituents of meteorites, such ages frequently represent mineral cooling ages that can postdate planetesimal formation by millions or tens of millions of years, depending on the cooling history of individual planetesimals at different depths. Nevertheless, such cooling ages provide a detailed thermal history which can be fitted by thermal evolution models that constrain the formation age of individual parent bodies. Here we apply state-of-the-art thermal evolution models to constrain planetesimal formation times particular in the outer solar system formation region of C meteorites. We infer a temporally distributed accretion of various parent bodies from $<0.6$ Ma to $\approx 4$ Ma after solar system formation, with 3.7 Ma and $2.5-2.75$ Ma for the parent bodies of CR1-3 chondrites and the Flensburg carbonaceous chondrite, and $<0.6$ and $<0.7$ Ma for the parent bodies of differentiated achondrites NWA 6704 and NWA 011, respectively. This implies that accretion processes in the C reservoir started as early as in the NC reservoir and were operating throughout typical protoplanetary disk lifetimes, thereby producing differentiated parent bodies with carbonaceous compositions in addition to undifferentiated C chondrite parent bodies. The accretion times correlate inversely with the degree of the meteorites’ alteration, metamorphism, or differentiation. The accretion times for the CM, CI, Ryugu, and Tafassite parent bodies of 3.8 Ma, 3.8 Ma, $1-3$ Ma, and 1.1 Ma, respectively, fit well into this correlation in agreement with the thermal and alteration conditions suggested by these meteorites. Our results indicate that individual planetesimals formed rapidly (i.e., within $<1$ Ma), however, distinct planetesimals formed recurrently throughout the total lifetime of the protoplanetary disk. Rapid individual formation is consistent with streaming instabilities assisted by gravitational collapse. However, obviously not the total dust inventory was consumed at early disk evolution stages, so there must have been some delay mechanisms, e.g. collisional destruction of precursor aggregates due to high impact velocities induced by radial drift phenomena. This counterbalance enabled late ($>2-3$ Ma) accretion of C planetesimals beyond the snow line which escaped severe planetesimal heating and volatile loss, hence, preserving their volatiles, especially water. Only this delayed formation of water-rich planetesimals allowed Earth to accrete sufficient water to become a habitable planet, preventing it from being a bone dry planet.

## Introduction

Accretion processes in protoplanetary disks produce a diversity of small bodies. In our solar system, small bodies played a crucial role in both early and late accretion of planets, after potentially multiple scattering of planetesimals at various heliocentric distances^1^.

Several lines of evidence based on studies of meteorites have been used to estimate the accretion timescales of their parent bodies and by inference the general formation times of both planetesimals and planets in the early solar system.

Formation of specific meteoritic components called chondrules occurred by flash-heating events of mm-sized objects in the solar nebula and, therefore, pre-dated the formation of the respective meteorite parent bodies. Chondrule ages derived from the application of radioisotope chronology estimate, therefore, the earliest possible parent body formation time^2^. With this approach, the latest accretion time is limited only by the dissipation of the protoplanetary disk, resulting in a potential error of several millions of years (Ma).

Another line of evidence are the ^182^Hf-^182^W isotopic compositions of iron meteorites, which have been interpreted as reflecting the timing of metal-silicate segregation, e.g., during formation of a planetary Fe-Ni core. Assuming an instantaneous core formation, timing of metal melting temperatures derived from numerical models has been compared with those ages, and parent body accretion times have been suggested^3,4^. Such an approach is afflicted by serious assumptions and simplifications, as demonstrated by Neumann et al.^5^ for the IVB iron meteorites. Potential errors of this approach on the accretion time are also on the order of magnitude of 1 Ma.

Some upper limits of parent body accretion time can be derived from ages of minerals that formed by aqueous or thermal activity on the parent body. It is important to note that these ages frequently resemble cooling ages that postdate parent body formation by a few or occasionally tens of millions of years. Nevertheless, they can be used to reconstruct the thermal history inside plantesimals and— provided that the applied thermal models are adequately sophisticated—to infer respective planetesimal accretion times. Such modeling approaches including $\chi ^2$ minimisation fit procedures of thermal history constraints were presented for ordinary chondrite parent bodies^6–11^ taking into account the porosity evolution during thermally induced sintering and compaction, and its influence on thermal conductivity. These models were also applied to primitive achondrites, carbonaceous chondrites, and the NEA Ryugu^12,13^. For differentiated parent bodies, such models consider metal-silicate separation, including formation of differentiated layers, such as Fe-Ni core and silicate mantle, magma ocean evolution, and liquid-state convection^5,12,14–16^.

In this paper, we apply above outlined modeling approaches to constrain the sequence of planetesimal formation in a confined outer region of the solar protoplanetary disk from which CR chondrites and compositionally similar meteorite parent bodies originate.

It is commonly accepted that meteorite parent bodies formed over some millions of years at different locations of the disk that caused their strongly diverse chemical and isotopic composition. However, it is a yet undecided question if planetesimal formation persisted at any time in any parts of the disk. A confined formation region of CR chondrite and related parent bodies is indicated by isotopic and chemical similarities which are different from other meteorite groups: First, in a plot of O versus Cr (or Ti) isotopic composition (supplementary material, Supplementary Figure 1), a dichotomy between non-carbonaceous (NC) and carbonaceous (C) meteorites is defined by different contributions of nucleosynthetic isotope anomalies^17^, besides the well-known differences in major, minor and trace element chemistry^18^. While this fundamental dichotomy is sometimes interpreted as reflecting formation locations inside and outside the region where Jupiter formed, it likely furthermore indicates accretionary processes reflecting water-poor and water-rich compositions inside and outside the early solar system’s snowline.

Parent bodies of some achondrites, i.e., differentiated meteorites, obviously accreted early and mostly in the NC region. By contrast, late accretion in the C region is believed to have produced mostly undifferentiated parent bodies. Although existence of carbonaceous iron meteorites has been interpreted as indicative of a relative early accretion, formation timescale of their carbonaceous parent bodies is a matter of an ongoing debate and of recent revisions towards a late accretion^19^.

We focus on a class of planetesimals that we may call the “CR meteorite clan” which can be distinguished not only from NC meteorites, but also from other groups of C meteorites (CI, CM, CO, CV, CK, and C2-ungrouped Tagish Lake) by a confined O and Cr isotopic composition in suppl. Fig. 1. The CH and CB groups are frequently considered to form a common clan with CR chondrites, but they are beyond the scope of the present study. The members of this clan considered here are the CR1-3 chondrites, Tafassites, NWA 6704 and NWA 011 grouplets, and, potentially, the C1-ungrouped carbonaceous chondrite Flensburg. While the similar nucleosynthetic isotopic composition implies a similar formation region, other geochemical and mineralogical properties indicate different parent bodies (see below).

The CR chondrite group was initially defined by the aqueously altered undifferentiated CR2 chondrites, later supplemented by CR1 and CR3 chondrites, where the numbers reflect the petrologic type, i.e., the alteration degree^20^. A separate group is formed by the ungrouped achondrite Tafassasset, the meteorites previously termed CR6 and CR7^21,22^, and further previously ungrouped meteorites ranging from metamorphosed chondrites to primitive achondrites^15^. This is the first established carbonaceous primitive achondrite group with the proposed name as Tafassites^15^.

In addition to Tafassites, further meteorites have been shown to be geochemically similar to CR chondrites, to have broadly similar oxygen isotope^15^ and chromium isotope compositions^23^, and, thus, to have likely formed from a related material^15^. Those are the basaltic achondrite grouplets defined after the ungrouped achondrite meteorites NWA 011^24^, NWA 6704^22^, and NWA 7680^23^, and, potentially, the C1-ungrouped chondrite Flensburg^25^.

While common oxygen and chromium isotopic composition of all these groups and grouplets indicate parent body formation in a confined C or CR like reservoir of the protoplanetary disk^15,26^, other geochemical indicators demand distinct parent bodies, e.g., incompatible element chemistry, FeO/Mn compositions, silicate FeO content, as well as further major, minor, and trace element compositions (see also supplementary material, Supplementary Figure 1 for oxygen isotope compositions)^15,25,27^.

Therefore, the broad geochemical, O isotope, and Cr isotope similarities indicate that Tafassites, NWA 6704, NWA 011, NWA 7680, Flensburg, and CR chondrites derive from at least six different parent bodies that formed from a similar material and within the same confined sub-reservoir of the C reservoir. With the exception of Flensburg, the term “CR clan meteorites” is appropriate for referring to those meteorites and we use this term for the CR chondrites, Tafassites, NWA 011, NWA 6704, and NWA 7680^23^. The finding of different meteorite groups and grouplets that represent different parent bodies, but sourced from the same sub-reservoir of the disk material, motivates a detailed model of parent body thermal evolution in order to determine their size and accretion time.

In addition to the CR1-3 chondrites, the only carbonaceous extraterrestrial materials that contain notable amounts of water are CM, CI, and Tagish Lake (TL) chondrites as well as CI-like Ryugu samples. Particularly the CI, TL, and Ryugu samples are chemically, mineralogically, and isotopically similar to each other and more similar to the CR chondrites than all other carbonaceous samples^28–31^. This suggests that they might source from a common sub-reservoir that was neighboring that of the CR clan samples in the protoplanetary disk and was spatially closer to it than the CO, CK, and CV sub-reservoirs.

Parent bodies of meteorites that had an initially water-rich composition have been suggested to have formed in the C reservoir beyond the orbit of Jupiter^17,32,33^. The striking contrast in the metamorphism and alteration degree of the CR clan members could imply substantially different maximum temperatures on their parent planetesimals^34^. This indicates potentially that the accretion times of these bodies could be scattered over several millions of years, since the strength of the heating can be approximated in a first-order approach with different concentrations of ^26^Al (with a half-life of 0.717 Ma) available at different times after the formation of the Ca–Al-rich inclusions (CAIs)^34^.

For the present study, we modeled parent bodies of the CR chondrites, Flensburg, NWA 011, and NWA 6704. The Tafassites parent body was already considered in our earlier study^15^, and for NWA 7680 not enough thermo-chronological data are available for modeling at present. For the broader context of more water-rich C parent bodies, we compare here also with our previous results for the parent bodies of the CI and CM chondrites and for the parent body of Ryugu^13^.

Multiple chronometers can provide precise age data for mineralogical components, that formed at elevated temperatures in the interiors of already existing planetary objects. Their formation times post-date the accretion times of the parent bodies and describe time-temperature paths that constrain parent body formation times. Carbonates that formed during the hydrothermal activity experienced by the aqueously altered meteorites incorporate Mn. Thus, the short-lived decay system ^53^Mn-^53^Cr with a ^53^Mn half-life of 3.7 million years can be used to constrain the carbonate ages. Heavily metamorphosed or partially molten meteoritic materials lack carbonates. Thus, other components, such as whole-rock, olivine, pyroxene, plagioclase, and phosphates can be dated using the U/Pb-Pb, Al–Mg, and Mn–Cr chronometers^15,16^. Strictly speaking, age data yield the cooling time below a critical closure temperature, below which daughter radionuclides cannot be diffusively exchanged between the dated mineral and the surrounding mineral matrix. Hence, different ages and closure temperatures yield cooling curves in the planetesimal’s interior.

Parent body accretion ages are very different from igneous crystallization ages of meteorites and they are model-dependent on the initial conditions of accretion, composition, radiogenic heating, water content, porosity etc. No accretion ages were reported for the NWA 6704 parent body, and only outdated model accretion ages for NWA 011 and Tafassasset that were proposed in Sugiura and Fujiya^35^ based on limited chronological records of these meteorites published at this time. CR clan achondrites, primitive achondrites, and chondrites have been interpreted by multiple workers as samples from partially differentiated planetesimals (e.g., Bunch et al.^36^, Wittke et al.^37^, Weiss and Elkins-Tanton^24^, Jiang et al.^38^). In Ma et al.^15^, we showed that CR6-7 chondrites cannot be from the same parent bodies as CR1-3 and CR-clan achondrites. Here, by fitting thermo-chronological data using state-of-the-art global thermal evolution and differentiation models for early solar system planetesimals^12,15^, we constrained ranges for the radii and accretion times appropriate for individual parent bodies that are likely to have produced various CR clan materials.

## Data and methods

Characteristic properties that we consider as the most important ones for the alteration, metamorphism, and differentiation of the parent body material are primarily the parent body size and accretion time, and secondarily its interior structure, including the burial depths of meteorites. These properties were derived here from the analysis of formation of various primary (olivine, pyroxene, plagioclase) or secondary (carbonates, such as calcite, dolomite, or breunnerite, formed in the presence of aqueous solutions) mineralogical components, or whole-rock samples (Table 1). While we present here calculations for four parent bodies, we provide in the following an overview of the data for all groups and grouplets discussed in the paper.

Carbonates formed close to the peak of hydrothermal activity on the parent bodies of CR, Flensburg, CI, and CM chondrites. Data available for CR chondrites give rise to Renazzo dolomite and calcite, and GRO 95577 calcite data points. Calcite and dolomite formation in Flensburg has been dated with almost indistinguishable relative formation times that yield a single data point^25^. The CI and CM carbonate formation ages have been summarized to three data points in Neumann et al.^13^. The CI dolomite ages are indistinguishable for different meteorites and give rise to a CI dolomite data point. In addition, protracted CI breunnerite formation has been dated. As in the case of CI dolomite, indistinguishable CM calcite ages produce one CM calcite data point.

In the heavily metamorphosed and partially molten Tafassites and differentiated NWA 011, NWA 6704, and NWA 7680 grouplets that lack carbonates, whole-rock, olivine, pyroxene, plagioclase, or phosphates were dated with the U/Pb-Pb, Al–Mg, and Mn–Cr chronometers (Table 1). The Tafassite U/Pb-Pb phosphate data were derived recently^15^, providing one data point for each of three North-West Africa Tafassites and Tafassasset in addition to previously available Hf–W and Mn–Cr data, while for the differentiated CR clan grouplets defined by NWA 011, NWA 6704, and NWA 7680, Al–Mg, Mn–Cr, or Pb–Pb data are available (Table 1).

Overall formation temperatures for all components in Table 1 are bracketed by $\approx 300$ K and $\approx 1300$ K, and the ages available fall into the first $\approx 27$ Ma after the solar system formation.

Planetary bodies that accreted within the time period covered by these data experienced an initial heating phase, mainly due to the decay of ^26^Al, followed by a protracted cooling phase after reaching a temperature maximum. Thus, the data describe the cooling behavior within the parent bodies at the burial depths of the meteorites after reaching a temperature maximum, except for Flensburg, where carbonates formed during the initial heating phase on the ascending branch of a temperature curve^25^. The carbonate ages in CI and CM meteorites fit to the above time scale^39^ providing a possibility for a comparison of parent body aqueous alteration history^13^. Note that in case of carbonates we used formation temperatures to fit the thermal history. Carbonate formation temperatures are known to be relatively low. We implicitly assume that at the low metamorphic temperatures affecting CR or Flensburg chondrites, the Mn–Cr system in carbonates was not open to diffusion, but that the formation process was the one starting the radioisotopic clock. Carbonate formation can occur on both the ascending or the descending temperature branch of thermal evolution, in contrast to the cooling ages of the other isotopic systems in Table 1.

**Figure 1:**
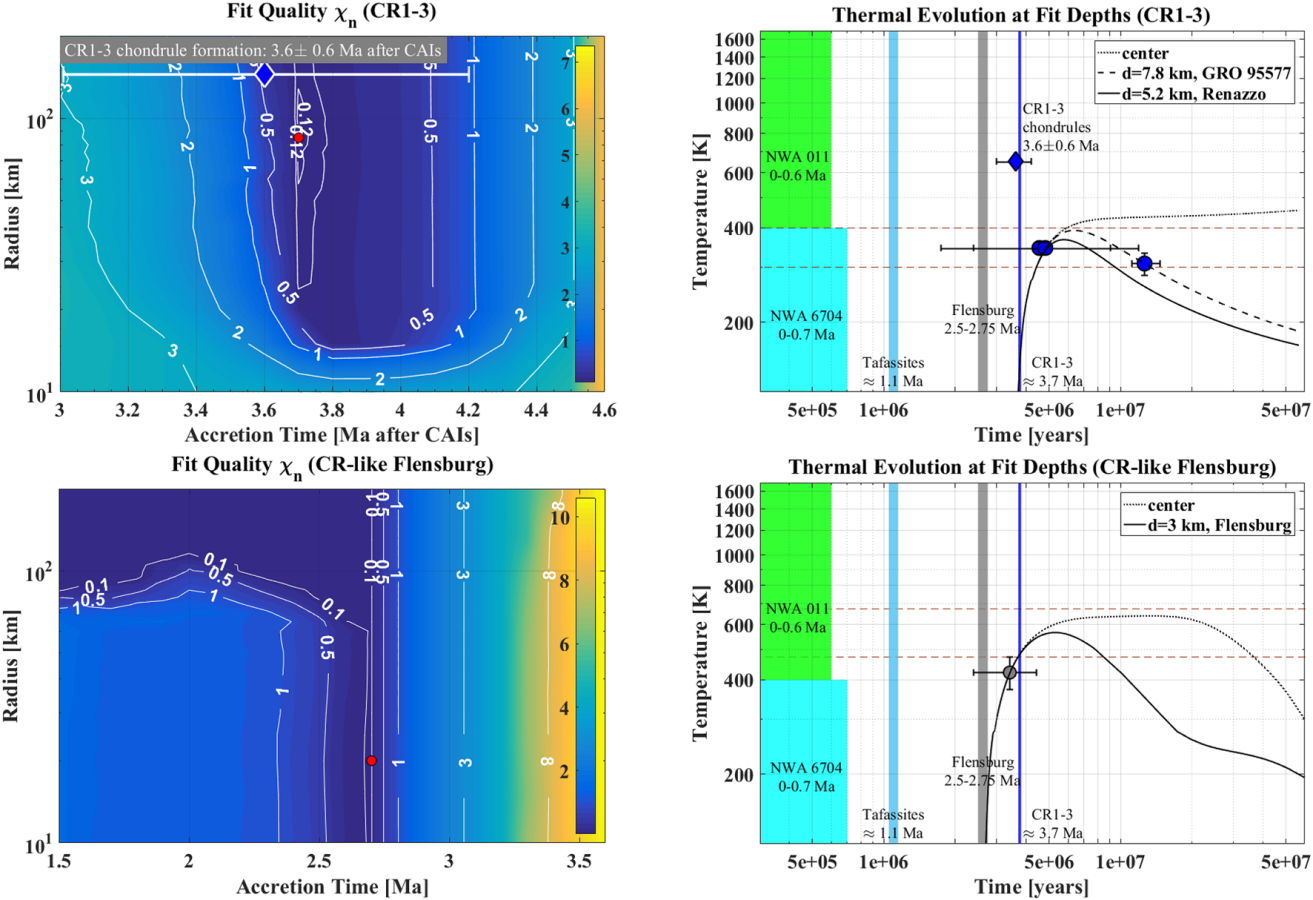
Left column: Both colorbar and isolines show fit quality as a function of planetesimal radius *R* and accretion time $t_{0}$ for CR1-3 chondrites (first row) and the Flensburg chondite (second row). Best-fit accretion times correspond from top to bottom to $\approx 3.7$ Ma and $\approx 2.5-2.75$ Ma. The exemplary parent bodies from the respective minimum fit quality regions with $R=90$ km and $t_{0}=3.7$ Ma and $R=20$ km and $t_{0}=2.7$ Ma, from top to bottom, are indicated with red dots. Right column: Thermal evolution at the best-fit depths *d* of respective meteorites for representative best-fit parent bodies indicated by red dots in the left column. Dashed red lines show the maximum metamorphic temperature estimates from petrological constraints (see also supplementary material). Color patches show PB accretion time intervals. The accretion time of the CR clan Tafassite PB is included for completeness.

The carbonate oxygen isotopic data (supplementary material, Supplementary Figure 1, top right panel) indicate similar carbonate formation conditions for CR, CI, CM, and TL, but not for Flensburg, in agreement with a higher alteration temperature suggested for the latter^25^. The differences between the data imply different alteration conditions, e.g., the duration, temperature, redox conditions, and fluid composition experienced^40^. These conditions can be reviewed using parent body thermal evolution modeling.

**Table 1:** Time (in Ma rel. to CAIs) and temperature (in K) data used for fitting the CR clan meteorite parent bodies.

Meteorite	Closure/formation *T*		Closure/formation *t*		Method
	$T^{c}$	$\sigma _{T}$	$t^{c}$	$\sigma _{t}$	
	K	K	Ma	Ma	
Aqueously altered CR chondrites					
Renazzo ^(Dol)^	344.5	16.5	4.8	+4.24/-2.4	Mn-Cr
Renazzo ^(Cal)^	344.5	16.5	4.55	+7.4/-2.8	Mn-Cr
GRO 95577 ^(Cal)^	308	25	12.57	+2.1/-1.4	Mn-Cr
Aqueously altered Flensburg chondrite					
Flensburg ^(Car)^	423	50	3.4	1	Mn-Cr
Aqueously altered CI chondrites					
Orgueil, Ivuna, Y980115 ^(Dol)^	398	50	5.485	0.655	Mn-Cr
Orgueil ^(Bre)^	398	50	9.7	3.6	Mn-Cr
Aqueously altered CM chondrites					
Murchison, Y791198 ^(Cal)^	318	50	5.445	0.655	Mn-Cr
Partially molten CR clan Tafassites					
Tafassasset ^(W)^	1200	100	2.9	0.9	Hf-W
Tafassasset ^(W)^	950	100	4.9	0.3	Mn-Cr
Tafassasset ^(Pho)^	720	50	19.86	8.1	U/Pb-Pb
NWA 11561 ^(Pho)^	720	50	7.55	2.85	U/Pb-Pb
NWA 7317 ^(Pho)^	720	50	8.42	2.94	U/Pb-Pb
NWA 12455 ^(Pho)^	720	50	10.1	1.75	U/Pb-Pb
Differentiated CR clan grouplets					
NWA 011 ^(Pl, Px)^	750	130	4.5	0.33	Al-Mg
NWA 011 ^(Ol, Px)^	850	100	5.62	2.64	Mn-Cr
NWA 2976 ^(W, Pl, Px)^	750	130	5.08	0.05	Al-Mg
NWA 2976 ^(Pl, Px)^	770	50	5.01	0.59	U/Pb-Pb
NWA 4587 ^(W, Pl, Px)^	750	130	4.68	0.04	Al-Mg
NWA 4587 ^(Px)^	770	50	5.47	0.23	U/Pb-Pb

NWA 6704 ^(Pl, Px)^	750	130	5.31	0.13	Al-Mg
NWA 6704 ^(Pl, Px)^	850	100	6.24	0.81	Mn-Cr
NWA 6704 ^(Px)^	770	50	5.14	+0.3/-0.22	U/Pb-Pb
NWA 10132 ^(Pl, Px, Ol)^	750	130	5.42	0.7	Al-Mg
NWA 7680	850	100	5.53	0.34	Mn-Cr

The data for water-rich CI and CM chondrites and for CR clan Tafassites are shown as well. Notes: ^(Bre)^ breunnerite, ^(Cal)^ calcite, ^(Dol)^ dolomite, ^(Pho)^ phosphate, ^(Pl)^ plagioclase, ^(Px)^ pyroxene, ^(W)^ whole-rock, ^(Ol)^ olivine, ^(Car)^ carbonates. The age data are from^39^(CR),^25^(Flensburg),^41^(NWA 011 and NWA 2976),^23,42^(NWA 4587, NWA 7680),^22,43^(NWA 6704),^23^(NWA 10132),^15,41^(Tafassites),^13,39^(CI, CM). The closure and formation temperatures are from^44,45^(CR1-3),^25^(Flensburg),^46,47^ and^48^(NWA 011, NWA 2976, NWA 4587, NWA 6704, NWA 10132),^15^(Tafassites),^13^(CI, CM). A CAI age of 4567.9 Ma was used.

The model we used to derive parent body properties^12,15^ calculates the thermal evolution for planetesimals heated by short- and long-lived radioactive nuclides. In the case of NWA 011 and NWA 6704, the models included metal-silicate separation, since melting and differentiation are indicated by the meteorites. The differentiation modeling included both percolation of metal and silicate melts at low-degree partial melting and separation by Stokes flow in the magma ocean regime, in agreement with the meteorite record and lab work^49^. Properties of the parent bodies were obtained by approximating the data points with the evolution of the temperature at different depths in the respective time interval using a root mean square (RMS) procedure and by comparing with the maximum metamorphic temperature estimates from petrological constraints^11,12,15^. By varying the radius *R* over a wide range of 10 km to 200 km and the accretion time $t_{0}$ between 0 and 5 Ma after CAIs, we constrained ranges within the $(R,t_{0})$-diagram appropriate for bodies that are likely to have produced various CR clan and related materials. This procedure determines best-fit depths for the meteorites within any planetesimal and parameter ranges for best-fit parent bodies with their respective best-fit depths for the meteorites. The numerical setup of the model including various effects of porosity evolution, latent heat of melting and crystallization for water ice, metal, and silicates, etc., is described in detail in the supplementary material.

## Results

**Figure 2:**
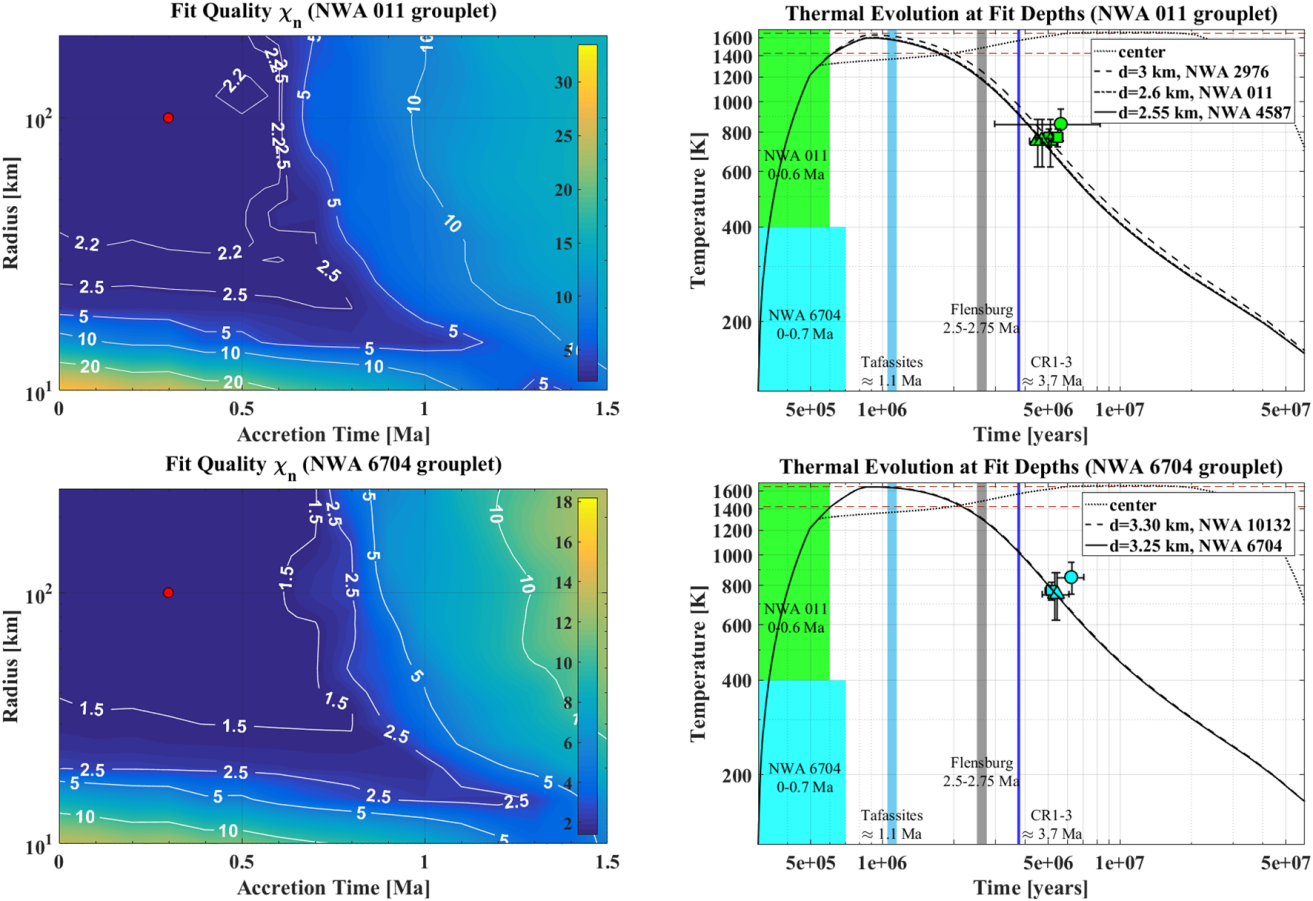
Left column: Both colorbar and isolines show fit quality as a function of planetesimal radius *R* and accretion time $t_{0}$ for the achondrites NWA 011 (first row) and NWA 6704 (second row). Best-fit accretion times correspond from top to bottom to $\lesssim 0.6$ Ma and $\lesssim 0.7$ Ma. The exemplary parent bodies from the respective minimum fit quality regions with $R=100$ km and $t_{0}=0.3$ Ma and $R=100$ km and $t_{0}=0.3$ Ma, from top to bottom, are indicated with red dots. Right column: Thermal evolution at the best-fit depths *d* of respective meteorites for representative best-fit parent bodies indicated by red dots in the left column. Dashed red lines show the maximum metamorphic temperature estimates from petrological constraints (see also supplementary material). Color patches show PB accretion time intervals. The accretion time of the CR clan Tafassite PB is included for completeness.

The fit quality $\chi _n$ value calculated (Figs. 1 and 2) shows best-fit parent body accretion times and sizes. The absolute value of the optimal fit quality is case dependent, while, in general, best-fit objects are characterized by its minimum. A global minimum is difficult to obtain given that the number of data points available varies between 1 and 6 for different parent bodies. Thus, a minimum or best-fit field defined in an intuitive manner by a plateau of the fit quality, and an acceptable fit quality defined by strong gradients around the boundaries of the plateau are more suitable than a global minimum. Conclusive constraints on the accretion time were obtained with overall uncertainties of $\lesssim 1$ Ma for each meteorite group, while the radius estimates are less ideal. For CR1-3, a field with a minimum fit quality $\chi _{n}\le 0.15$ is obtained for an accretion between 3.7 and 3.8 Ma after CAIs (Fig. 1, top left panel). The corresponding radius spread is $30-200$ km, but a minimum of 0.12 for $\chi _{n}$ is obtained at $t_{0}\approx 3.7$ Ma and $R=70-120$ km. For an acceptable fit quality of $\le 0.5$, the accretion time is bracketed by 3.6 and 4.1 Ma. Overall, the best-fit accretion time of 3.7 Ma is almost indistinguishable from the CR1-3 chondrule formation time of $\approx 3.6\pm 0.6$ Ma^2,50^.

In the case of Flensburg, an acceptable $\chi _{n}\le 0.5$ field is obtained between $t_{0}=2.5$ and $t_{0}=2.75$ Ma for any radius, but also for an accretion as early as 1.5 Ma of bodies larger than $R=80$ km (Fig. 1, second row, left panel). Thus, no constraint on the radius can be derived from the fit procedure. The reason for that and for an acceptable fit quality for an early accretion is the availability of only one data point. For any object within the field with $\chi _{n}\le 0.5$, a depth can be found at which the temperature curve crosses the center of the data point on the ascending branch, consistent with the prograde carbonate formation in Flensburg. However, the earlier the accretion, the shallower this depth (down to $\lesssim 300$ m) and the higher the peak central temperature of the planetesimal, correlating inversely with the accretion time and exceeding the melting temperature in extreme cases. Thus, we consider planetesimals that accreted prior to 2.5 Ma as less likely parent body candidates. Those that accreted between 2.5 and 2.75 Ma have, by contrast, mostly homogeneous structures and thermal conditions throughout their interiors, in agreement with the prevalent notion of water-rich chondrite parent bodies. An accretion time of $\approx 2.7$ Ma fits the Flensburg carbonate data best for any parent body size.

The fit for the achondrites NWA 011, NWA 2976, and NWA 4587 (Fig. 2, top left panel) produced an acceptable fit quality of $\chi _{n}\le 2.5$ for an accretion between 0 and 0.6 Ma and radii of $>20$ km. A minimum field can hardly be identified, if at all, by $\chi _{n}\le 2.2$ that does not affect the accretion time interval, but changes the radius estimate to $>40$ km. Relatively high fit quality values are caused by a maximum time difference of $\approx 1$ Ma at the depth of NWA 4587, very small uncertainties of Al–Mg ages of 0.33, 0.05, and 0.04 Ma for NWA 011, NWA 2976, and NWA 4587, respectively, and a necessity to fit each of these data points along with the respective Mn–Cr or Pb–Pb data at the same depth for each meteorite.

Finally, the fit for the grouplet comprising NWA 6704 and NWA 10132 was done with four data points. The shapes of the $\chi _{n}$ isolines (Fig. 2, bottom left panel) resemble those obtained for the NWA 011 grouplet, as it was expected by the relative similarity of the data. Models produced an acceptable fit quality of $\chi _{n}\le 1.8$ for $t_{0}\lesssim 0.9$ Ma and $R>20$ km, and a plateau with $\chi _{n}\le 1.5$ for an accretion before 0.7 Ma and a radius of $>30$ km.

The right columns of Figs. 1 and 2 show temperature curves at best-fit burial depths of the meteorites from which the data were obtained within the representative best-fit parent bodies. The latter are indicated by red dots in the respective fit quality panels (left columns). For CR1-3 (Fig. 1, top right panel), two depths corresponding to two meteorites Renazzo (5.2 km) and GRO 95577 (7.8 km) were calculated within an object with a radius of 90 km. The temperature curves fit the Mn-Cr data on descending branches well within the uncertainties and the maximum values of 370 K or 395 K agree with the temperature ranges suggested from lab work and with the petrologic type. A larger burial depth and a longer heating phase of GRO 95577 are in agreement with its high alteration degree compared to Renazzo and corroborate, further, the positions of these meteorites on the CR mixing line as a result of their alteration extents. While, in principle, for slightly earlier accretion times temperature curves can be obtained that descend right between the centers of the Renazzo data points, then the GRO 95577 data point will fitted with a poor fit quality that would make the overall parent body fits worse than in the minimum field at $t_{0}\approx 3.7$ Ma.

From one data point available for Flensburg, only one burial depth within the parent body could be fitted (Fig. 1, bottom right panel). The temperature curve for the representative body with $R=20$ km crosses the Mn–Cr carbonate data point on its ascending branch in agreement with the data point reflecting the age of an early and prograde carbonate formation and the lack of carbonate forming fluids later on. A maximum temperature of 565 K reached lies well within the interval of $470-670$ K suggested^25^.

In the case of NWA 011 grouplet (Fig. 2, top right panel), three meteorites with two data points each resulted in fits of three different burial depths within the parent bodies in the $\chi _{n}$ minimum field. Burial depths of 2.55, 2.6, and 3 km within a body with $R=100$ km correlate with the decreasing Al–Mg age of the meteorites, reflecting stronger heating and slower cooling at higher depth. A burial depth difference of $\approx 50$ m for NWA 011 and NWA 4587 results from an Al-Mg age difference of only 0.18 Ma. The maxima of the respective temperature curves of $1610-1630$ K are sufficient to produce basaltic melts consistent with the composition of the grouplet members and our model produced differentiated silicate material at the fit depth. However, depths of $2.3-3$ km within the sample parent body also implies non-extrusive in situ formation for these meteorites.

For the NWA 6704 grouplet, fits of three NWA 6704 and one NWA 10132 data points produce typically two different depths (Fig. 2, bottom right panel). Both fits are dominated by the Al-Mg age difference of 0.1 Ma that resulted in a burial depth difference of $\approx 50$ m. A deeper location for NWA 10132 with an younger Al-Mg age is consistent with slightly higher temperature maximum and slower cooling. Similarly to the NWA 011 grouplet, maximum temperatures of $1645-1648$ K agree with silicate melting, while the differentiated silicate material obtained with our model at the burial depths agrees with the meteorite composition. Burial depths of $\approx 3.3$ km imply an non-extrusive in situ formation also for this grouplet.

**Figure 3:**
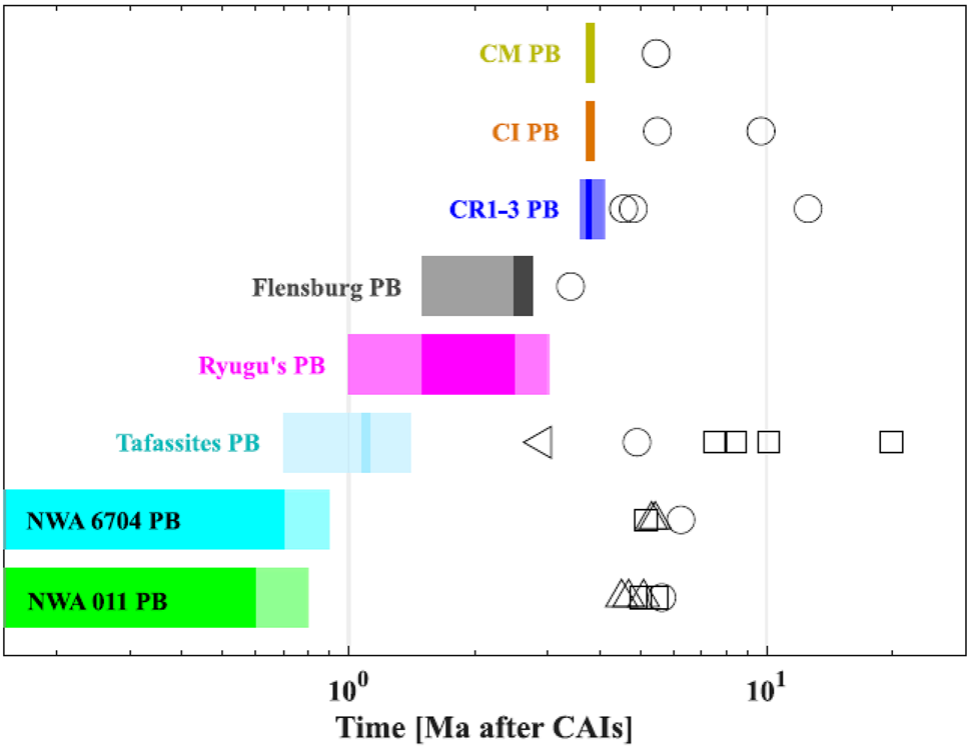
Parent body accretion times (colored patches, dark for best-fits and transparent for acceptable fits) and meteorite metamorphic ages (data points) for the C reservoir modeled incl. Tafassites, Ryugu, CI, and CM PB^13,15^. Younger Mn-Cr carbonate ages (circles) of CR1-3, CI, and CM than for Flensburg result in moderately younger accretion age for the CR1-3, CI, and CM PBs. NWA 6704 (cyan) and NWA 011 (green) have older Pb–Pb pyroxene and whole-rock ages (squares) than Tafassites (light blue), resulting in earlier accretion than the Tafassite PB. The NWA 011 and NWA 6704 Al–Mg (triangles) and Mn–Cr (circles) data support an early PB formation. While Tafassites^15^ also include a very late, i.e., young, phosphate Pb–Pb age, their old iron-silicate Hf–W (triangle) and Mn–Cr ages cause a shift to an intermediate PB formation time between NWA 011 and NWA 6704 on one hand, and Flensburg, CR, CI, and CM PBs on the other, while the young Pb–Pb age has its major effect in the large Tafassites PB size. All accretion times correlate inversely with the meteorite petrologic type and the degree of metamorphism or melting. See Table 1 for the thermo-chronological data.

## Discussion

Our calculations indicate an origin on different parent bodies for the meteorite groups considered, with a parent body accretion time of $t_{0}\approx 3.7$ Ma and size of $R \approx 90$ km for the CR1-3, $t_{0}\approx 2.5-2.75$ Ma for Flensburg, $t_{0}\lesssim 0.6$ Ma and $R>40$ km for NWA 011 grouplet, and $t_{0} \lesssim 0.7$ Ma and $R>30$ km for NWA 011. This demonstrates a spread of formation times between 0 Ma and 4 Ma after the formation of the solar system in the accretion disk region of carbonaceous chondrites. Accretion times we derived recently for other (initially) water-rich parent bodies, such as those of Tafassites ($1.1_{-0.4}^{+0.3}$ Ma)^15^, Ryugu ($1-3$ Ma)^13^, CM, and CI (both $\approx 3.8$ Ma)^13^ support this conclusion. Our results confirm, further, that rocky parent bodies accreted sequentially^15^ from 0 to 4 Ma after CAIs, and witnessed different accretion conditions within a limited region of the outer solar system. This accretion scenario is different from those proposed earlier, of an early accretion and partial differentiation or of an early accretion, differentiation, and a late chondritic veneer^24,36,38,51^. Our results imply that there was an early accretion of fully differentiated CR-clan bodies, intermediate accretion of partially differentiated Tafassites, and late accretion of undifferentiated CR-clan or closely related chondritic bodies within the CR sub-reservoir of the protoplanetary disk.

The CR chondrite parent body complements the CI and CM parent bodies with a considerably smaller radius of $R\approx 15-25$ km but a similar accretion time of $3.75-3.8$ Ma and a sub-10 km sized parent body of the NEA Ryugu that accreted between 1 and 3 Ma suggested recently^13^. Our results for the CR chondrite parent body and the Tafassites parent body model^15^ support, further, that the meteorites classified initially as CR6-7 and re-classified as Tafassites do not belong to the same group as aqueously altered CR. An earlier formation of the NWA 011 and NWA 6704 grouplets’ parent bodies than that of the Tafassites is in an agreement with the former being differentiated and the latter only partially molten.

The parent body size estimates are less conclusive due to the lack of data but they fall into the range of typical sizes estimated for early solar system planetesimals^52^. Of note is a size contrast to the sub-10 km radius parent body of Ryugu that was derived based on porosity modeling^13^ and supported by later studies^53,54^.

Two types of internal parent body structures were obtained with respect to the degree of the metal-silicate differentiation— largely homogeneous vs. differentiated. Parent bodies of CR and Flensburg chondrites are homogeneous with maximum temperatures remaining below the melting temperatures of metal or silicates. Internal heating by ^26^Al warmed large fractions of their volumes relatively uniformly, leaving a small volume of outer layer relatively cool, and supporting production of petrologic types 1–3 at different depths. While the CR carbonate ages are younger than Flensburg’s within the margin of error, an earlier accretion of the Flensburg asteroid than the CR chondrite PB derived confirms that it recorded the earliest fluid activity on a planetary object in the solar system^25^. CR and Flenburg alteration conditions are similar to those of CI and CM PBs^13^, while bearing similarity to a lesser extent to the original parent body of Ryugu that was suggested to be heated to a higher temperature that enabled a partial dehydration^13,55^. In addition, peak central temperatures of up to 700 K obtained for the favored Flensburg PB models also come close to those derived for Ryugu from a potential partial dehydration.

With respect to the parent body structure and mechanical properties, the bulk porosity and porosity structure obtained for the CR and Flensburg chondrites here and for CI and CM chondrites by Neumann et al.^13^ are similar to each other, but different from those of the Ryugu parent body^13^. This contradicts a close compositional similarity to the CI material^30^ inferred by the Ryugu sample analysis and the suggestion of a potential origin on a common parent body. Ryugu samples originate from two different sites and have high porosities comparable with that derived by MASCOT that observed at yet another site. Thus, the asteroid is suggested to be homogeneous. However, an origin of high-porosity Ryugu material and low-porosity CI meteorites on a common parent body would require either a strong sorting of the rubble after parent body destruction, such that only high-porosity pieces would accrete to form Ryugu, but such a sorting appears unlikely. Or, it would require different impacts onto the parent body, of which one would be only shallow and eject enough material from a high-porosity surface layer to form Ryugu, and at least one other impact would eject CI material from deeper within where porosity has been reduced stronger.

An opposite differentiated structure type was produced due to partial melting in the interior of NWA 011 and NWA 6704 parent bodies. These structures are similar to those of primitive achondritic Acapulco-Lodran^12^ and Tafassites parent asteroids^15^. They are, further, consistent with the accretion times calculated for them and reported for achondrites and primitive achondrites in the literature. Not much chondritic material with typical petrologic types relative to the total parent body volume can be expected on these parent bodies due to small burial depths for of overall $2.5-3.3$ km derived for the differentiated meteorites. By contrast, primitive achondritic and achondritic materials are abundant at these depths and further below. If not removed totally by impacts, any kind of chondritic material could be retained only in thin surface shells, while most of the parent body volume would be dominated by achondritic metal or silicate rocks.

In particular, early accretion of the NWA 011 and NWA 6704 parent asteroids implies a similar magmatic evolution to Vesta that formed within $\lesssim 1$ Ma^14^. Bearing similarities to eucrites and classified originally as a non-cumulate eucrite^56^, NWA 011, and also NWA 6704 likely experienced similar petrogenesis. Given overall burial depths of $2.55-3.25$ km derived, both grouplets would be non-extrusive rocks that formed in situ and, in fact, NWA 011 has a cumulate-eucrite-like REE pattern^57^, while NWA 6704 spectrum showed a close similarity to the basaltic and cumulate eucrites^58^.

The asteroid (34698) 2001 OD22 suggested as parent body of NWA 6704^59^ does not fulfill the size requirements we derived and, if related, should be a piece of a once larger, now fractured parent asteroid. If (34698) 2001 OD22 is not related to NWA 6704, then it could be a primordial planetesimal, since collisional lifetimes of similar size objects correspond to the age of the solar system^60^). A partially differentiated parent body with a radius of $\approx 200$ km has been proposed previously as parent body of the CR group and NWA 011 grouplet^36,61,62^. Our results contradict that, both due to a smaller best-fit CR chondrite parent body size and a substantially earlier formation time of the NWA 011 parent body.

A lack of sufficiently large asteroids with exposed silicates that would meet compositional constraints of CR clan meteorites suggests that their parent bodies were removed from the asteroid belt or have experienced mantle removal and their cores are represented by metallic asteroids. As an tentative example, the presumably metallic asteroid (16) Psyche has a current maximum radius of $\approx 140$ km. Its initial radius including the lost silicate portion should have been at least 200 km. Hence, the initial body would be consistent with the best-fit fields for NWA 011 and NWA 6704. Regardless of parent body removal or loss of mantle, ejection of mantle portions by large-scale energetic collisions could have produced small basaltic meteorite parent body fragments that are still present in the asteroid belt yielding meteorite-sized fragments upon small impacts. For the asteroid Psyche, other models exist to explain the metal enrichment on its northern hemisphere, particularly because of the rocky appearance of its southern hemisphere. Prestgard et al.^63^ discuss the possibility of surficial metal enrichment by ferrovolcanism which could cover an undifferentiated outer shell similar to CR chondrite material. If so, then Psyche’s present maximum radius could be very close to the original one and would overlap marginally with the upper value of our results for the acceptable size of the CR parent body.

Accretion times of CR clan parent bodies including the result for Tafassites^15^ with other water-rich parent bodies from the C reservoir, such as that of Ryugu and of CI and CM groups, are compared in Fig. 3. The emerged accretion trend is correlated with the petrologic type of the meteorites and the degree of metamorphism or melting in agreement with the control of the heating rate by the amount of ^26^Al incorporated. The result of a temporally distributed accretion of CR clan parent bodies implies that accretion processes in the C reservoir started as early as in the NC reservoir and produced differentiated parent bodies with carbonaceous chondritic compositions, along with a spectrum of petrographic types, compositions, and parent body structures. This implication is supported by disk evolution modeling that identified the conditions that may lead to the contemporary formation of iron meteorite parent bodies at two distinct radial locations in the disk^64^.

Additional constraints of the Ryugu parent body age were expected from analyses of samples returned by Hayabusa2, however, there are still conflicting results on Mn–Cr dating of carbonates, specificly dolomite. While McCain et al.^53^ suggest dolomite formation within 1.8 Ma after CAIs in a planetesimal with a radius of $<10$ km (which, therefore, accreted even earlier), Nakamura et al.^65^ infer 2.6 Ma and Yokoyama et al.^30^ infer 5.2 Ma after CAIs, allowing a later accretion than within 1.8 Ma and, potentially, a larger parent body radius than 10 km. Further constraints on another CI- or CM-like chondrite parent body is expected from ongoing analyses of asteroid Bennu samples recently returned by the OSIRIS-REx mission.

Generalizing our results for other C meteorite clans would suggest formation of each clan from its own stable sub-reservoir over extended periods of time. Such a generalization would require knowledge of multiple formation times for each of the different clans. For CI and CM clans, only the late accretion close to 4 Ma after CAIs has been confirmed, but the respective related (primitive) achondrites are lacking. Since Ryugu has a CI-like composition and has been suggested to share a common parent body with the CI chondrites (and, potentially, with Tagish Lake), its accretion time estimate of $1-3$ Ma^13,53,65^ extends the accretion and the reservoir stability time interval for the CI clan closer to CAIs. However, not enough material is available to infer stability time intervals for the sub-reservoirs of all the different C clans.

Our results indicate that planetesimal formation was possible throughout disk lifetimes in a distinct location and reservoir of the early solar nebula. This finding has important implications for planet formation theories. For many years, theories of planetesimal formation faced fundamental problems, most of them associated with the so-called meter-size barrier^66–68^. While particle growth from submicrometer-sized dust to cm sized aggregates was proven to work in laboratory hit-and-stick collisions at low impact velocities below 1 m/s, aggregate collisions at a few 10 m/s turned out to be destructive, thus, preventing further growth^66^. Such high collision speeds were inferred from radial drift of meter-sized bodies towards the protostar due to deceleration by the head wind of the surrounding gas orbiting the star with sub-Kepler velocity (e.g., Weidenschilling^67^, Birnstiel et al.^68^).

Present theories of planetesimal formation via streaming instabilities assume rapid planetesimal formation in locations of the protoplanetary disk, where dust to gas ratios exceed distinct threshold values^69–71^. Planetesimal formation via streaming instabilities with the help of self-gravity^69–71^ provides an effective solution to overcome the meter-size barrier sufficiently fast, without collisional destruction or loss of pebbles to the central star. While the rapid formation mechanism is consistent with our assumption of quasi-instantaneous accretion, the range of formation times over several Ma indicates that individual planetesimals can form at a distinct location recurrently throughout the life-time of the protoplanetary disk.

Our results imply that the counteracting mechanisms of occasional rapid growth on the one hand, and ongoing destruction of meter-sized bodies on the other hand, balance each other, which is not a matter of course. If streaming instabilities would be much more effective than destructional mechanisms, then all the dust material in a protoplanetary disk would be consumed rapidly into planetesimals, on a short time scale compared to the typical disk lifetime ($<6$ Ma^72^). In such a scenario, we may envisage protoplanetary disks with nearly all planetesimals having formed within $<1$ Ma. In such a disk with an inventory of short-lived ^26^Al similar as our own solar system, nearly all planetesimals would have heated up to differentiation inducing a severe degree of volatile loss.

Inner planets in such systems could fail to accrete sufficient water-bearing planetesimals from beyond the snow-line, which was likely the case for our Earth^73,74^. This allowed sufficient water on Earth’s surface to become a habitable planet. It requires a substantial portion of water-rich planetesimals outside the snow-line that formed relatively late, e.g. $>2-3$ Ma after solar system formation.

We can conclude that counteractive mechanisms like the meter-size barrier delayed planetesimal formation by streaming instabilities in the early solar system. In this way, planetesimal formation was possible throughout the disk lifetime, even in spatially confined regions, and was not limited to certain formation time intervals within specific spatially distinct regions. It is yet unclear if this kind of recurrent planetesimal formation occurs in other extrasolar systems in a similar way, which needs to be investigated by theoretical or observational approaches.

Direct observations of accretion in different parts of protoplanetary disks are now obtainable by facilities such as ALMA or JWST, additional analyses are achievable using observations by VLT, while exoplanet search projects, such as PLATO and CHEOPS telescopes, can provide further clues via statistical information on the architecture of planetary systems.

### Supplementary Information

Supplementary Information.

## Data Availability

The data that support the plots within this paper and other findings of this study are available from the corresponding author upon reasonable request.

## Code Availability

The numerical model used is described in detail in the supplementary material. The code is available only from the corresponding author upon reasonable request.
