# Supplementary material for: Recurrent planetesimal formation in an outer part of the early solar system
Source: Sci Rep. 2024 Jul 1;14:14017. doi: 10.1038/s41598-024-63768-4 (PMC11217279; doi:10.1038/s41598-024-63768-4)
Supplement: Supplementary file 1 — Supplementary Information. [file 41598_2024_63768_MOESM1_ESM.pdf]

Supplementary Material for "Recurrent planetesimal formation in an outer part of the early solar system"

Wladimir Neumann[1,2,3,*], Ning Ma[4], Audrey Bouvier[5], and Mario Trieloff[2]

[1]Institute of Geodesy and Geoinformation Science, Technische Universität Berlin, Kaiserin-Augusta-Allee 104-106, 10553 Berlin, Germany
[2]Klaus-Tschira-Labor für Kosmochemie, Institut für Geowissenschaften, Universität Heidelberg, Im Neuenheimer Feld 234-236, 69120 Heidelberg, Germany
[3]Institute of Planetary Research, German Aerospace Center (DLR), Rutherfordstr. 2, 12489 Berlin, Germany
[4]Institute of Geochemistry and Petrology, ETH Zürich, Sonneggstrasse 5, 8092 Zürich, Switzerland
[5]Bayerisches Geoinstitut, University of Bayreuth, 95440 Bayreuth, Germany
[*]Corresponding author, wladimir.neumann@dlr.de, orcid=0000-0003-1932-602X,
www.researchgate.net/profile/Wladimir_Neumann

ABSTRACT

This is supplementary information for the manuscript "Recurrent planetesimal formation in an outer part of the early solar system".

Isotopic Systematics

Meteorite nucleosynthetic anomalies distinguish two major groups of meteorites, NC and C (Supplementary Figure 1, bottom panel),[1] and two major reservoirs of planetesimal formation separated early on in the protoplanetary disk.

The CR clan meteorite groups formed by CR chondrites, Tafassites, NWA 011, NWA 6704, NWA 7680, and, potentially, the chondrite Flensburg, have C-like nucleosynthetic anomalies. Specifically, they plot closely to each other and to CH and CB chondrites in the O vs. Cr isotope plot (Supplementary Figure 1). The CB, CH and CR chondrites are metal-rich carbonaceous chondrites, often grouped together as the CR chondrite clan. All these meteorites are within the same C region highlighted in Supplementary Figure 1 and their parent bodies accreted, thus, in the C reservoir of the protoplanetary disk. Specifically, they accreted in the same relatively confined region, as indicated by their similar oxygen isotopic composition. More broadly, the isotopic and compositional properties of the CR clan groups reflect a similar material with other C meteorites, such as CI, CM, Tagish Lake, and with C-type NEAs like Ryugu and Bennu.

Meteoritic components provide evidence for the isotopical heterogeneity of the early solar nebula and carry ^{16}O excesses derived from nucleosynthesis or CO self shielding.[2] Isotopic variations from inter-reservoir exchange give rise to linear mixing lines for different meteorite groups or clans in the oxygen three-isotope plot.[3,4] A common original material of CR chondrites and CR clan meteorites termed previously as CR6 or CR7 but re-classified recently as primitive achondrites with Tafassites as a proposed group name.[5] is indicated by oxygen isotope systematics (Supplementary Figure 1, top panels). A striking contrast in the metamorphism and alteration degree, however, suggests distinct evolution paths and different parent bodies. Comparing with other water-bearing carbonaceous chondrites, the CR chondrite bulk oxygen isotope compositions form a unique mixing line[2,6] that requires an anhydrous component precursor different from CI and CM groups.[6,7] An oxygen isotopic heterogeneity within the CR group and patterns similar to CM imply low-temperature aqueous alteration on an isotopically heterogeneous chondritic parent body (PB).[2,8] The CR6-7 data are broadly similar to the CR chondrite mixing line, but give rise to a separate Tafassite mixing line.

Further meteorites geochemically similar to CR include the basaltic achondrite grouplets NWA 011,[9] NWA 6704,[10] and NWA 7680,[11] other members of the Tafassites group,[5] and, potentially, the chondrite Flensburg.[12] Precursors of NWA 011, NWA 6704 and NWA 7680 grouplets are related to CR chondrites based on their broadly similar oxygen isotope compositions. NWA 011, NWA 6704, and NWA 7680 are, further, related to CR based on the $\varepsilon^{54}Cr$ similarity.[11] Considered together with CR chondrites, these meteorite groups and grouplets are clearly distinct from other carbonaceous meteorites[5] and likely belong to the CR clan, with the exception of Flensburg. However, the NWA 011 grouplet likely does not originate from the same parent body as Tafassites, due to different FeO-Mn compositions and chemical composition, e.g., higher silicate FeO content and Ca-rich pyroxene. The NWA 6704 grouplet's Δ^{17}O values suggest yet another separate parent body. The Flensburg bulk oxygen isotope data plot in the transition field to CR chondrites with a marginal overlap.[12]

Therefore, given the differences mentioned above, the broad oxygen isotope composition similarity indicates that Tafassites, NWA 6704, NWA 011, NWA 7680, Flensburg, and CR chondrites derive from at least six different parent bodies that accreted from a similar material in a spatially confined sub-reservoir of the C reservoir of the protoplanetary disk.

More broadly, the oxygen isotope systematics of the CR clan members reflect a similar material with other water-bearing C meteorites, such as CI, CM, and Tagish Lake (TL) and with C-type NEAs like Ryugu and Bennu. The genetic relationship of CR clan meteorites and accretion from a similar material imply that their oxygen systematics were produced by an isotopic exchange between ^{16}O-rich solids and ^{16}O-poor gases.[3] In general, the bulk O isotope compositions of the CR clan meteorites plot in a more ^{16}O-rich region relative to CI, CM, TL, and Flensburg meteorites (Supplementary Figure 1, top left panel). Similar δ^{18}O and δ^{17}O values imply that all CR clan groups are derived from a single relatively homogeneous region of the solar nebula. This region contained more ^{16}O-rich material than the Earth and CI chondrites based on the position below the terrestrial fractionation line (TFL) that is characteristic for the bulk composition of the Earth.

A unique bulk O isotope mixing line of the CR chondrites[2,6] requires a precursor material different from CI, CM, and Tagish Lake[6,7], implying formation in different C sub-reservoirs. Other CR clan members either form unique mixing lines (Tafassites), or have oxygen isotope systematics different enough from CI, CM, and Tagish Lake to imply different precursor materials and support different sub-reservoirs.

The carbonate oxygen isotopic data indicate partially similar carbonate formation conditions concerning temperature, oxygen fugacity, and carbon source in CR chondrites as in CI, CM, and TL chondrites, but different to Flensburg (Supplementary Figure 1, top right panel). Both implications agree with roughly similar alteration temperatures for CR, CI, CM, and TL, but a higher alteration temperature of Flensburg. The differences in Δ^{17}O and δ^{18}O values between the groups imply different alteration conditions, e.g., the duration, temperature, redox conditions, and fluid composition experienced.[6]

Thermal Evolution Model

A 1D finite differences thermal evolution model for planetesimals heated mainly by ^{26}Al presented in[13],[14] and[15] was adapted for the current study. It calculates heating of small porous bodies, their thermal evolution and compaction of a mixture of dry and hydrated material from an initially unconsolidated state due to hot pressing, and metal-silicate differentiation, by solving a number of equations that describe these processes. The model description is provided in the following.

A non-stationary 1D heat conduction equation in spherical coordinates is discretized by the finite differences method along the spatial and temporal domain and solved for the temperature:

$$\rho c_p \left(1 + x_{\mathrm{H_2O},0} S_{ice} + x_{Fe} S_{Fe} + x_{\mathrm{Si}} S_{Si}\right) \frac{\partial T}{\partial t} = \frac{1}{r^2} \frac{\partial}{\partial r} \left(k r^2 \frac{\partial T}{\partial r} \right) + Q(r,t), \quad (1)$$

with the bulk density ρ, the heat capacity c_p, the initial fractions x and Stefan numbers S for water ice, metal (Fe), and silicates (Si, see Supplementary Table 1), the temperature T, the time t, the radius variable r, and the energy source density Q. Equation (1) starts with the same initial temperature T_S at any r. It uses a zero heat flux boundary condition at the center and a constant temperature boundary condition T_S at the surface of a planetesimal. The initial and surface temperature of 120 K is used based on[16] in agreement with the accretion of the CR parent body at > 5 AU[17]. The energy source for the temperature change is radioactive decay of typical short-lived radionuclides ^{26}Al and ^{60}Fe and long-lived ^{40}K, ^{232}Th, ^{235}U, and ^{238}U:

$$Q(r,t) = \rho \sum_i f_i Z_i \frac{E_i}{\tau_i} \exp\left(-\frac{t - t_0}{\tau_i}\right), \quad (2)$$

with the porosity-dependent bulk density ρ, the number of atoms of a stable isotope per 1 kg of the primordial material f, the initial ratio of radioactive and stable isotope Z, the decay energy E, the mean life $\tau = \lambda / \log(2)$, the half-life λ, and the accretion time t_0 of the planetesimal. See Supplementary Table 2 for the associated parameter values. A homogeneous heat source distribution within the material is assumed, while the heat source density scales further with the porosity ϕ. The porosity is initially constant throughout the interior, but develops inhomogeneously with depth during the thermal evolution under the action of temperature and pressure. If metal and silicates separate, the summands in Eq. (2) are multiplied with the local ratio of the current volume fraction to the initial volume fraction of metal for ^{60}Fe or of silicates for all other nuclides involved.

Fitting Procedure

An approach utilized in several H and L chondrite parent body studies (e.g.,[18,19]) and a study of the Acapulco-Lodran parent body[20] is used to fit the thermo-chronological data with a least square procedure. The goal of the fitting procedure is to find model parameters that result in a thermal evolution model for the parent body which reproduces the thermo-chronological data of a CR-like meteorite group as close as possible. For this, a thermal evolution model is calculated for a given set of model parameters. The meteorites can be clustered according to their pairings or further criteria, such as the affiliation with a subgroup,

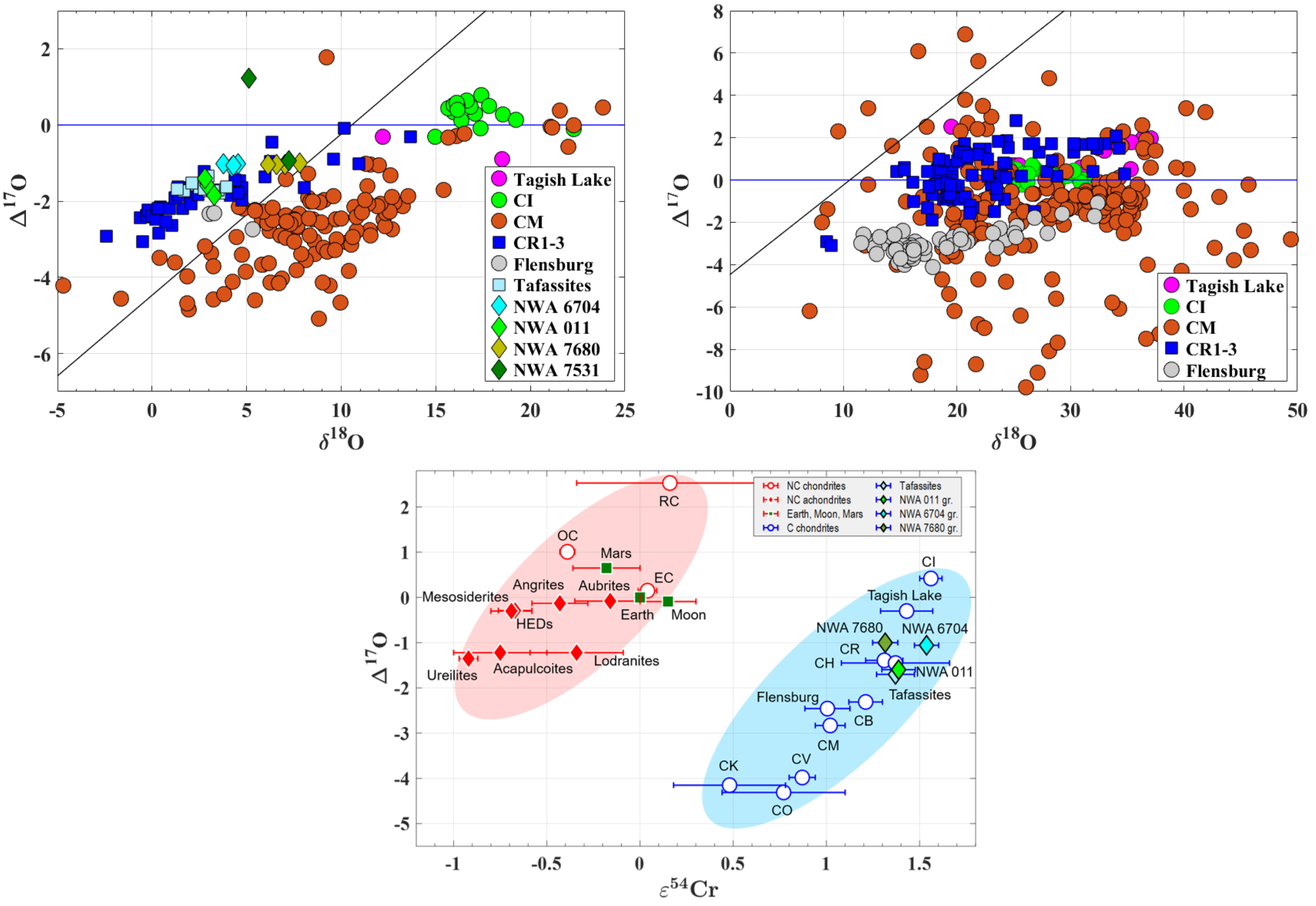

Supplementary Figure 1. Bulk (*top left*) and carbonate (*top right*) oxygen isotope data of CR clan meteorites and Flensburg in comparison with CM, CI, and Tagish Lake. *Bottom*: Comparison of the Δ^{17}O-ε^{54}Cr systematics of CR clan carbonaceous achondrites with other C and NC materials. The oxygen isotope data were obtained from the Meteoritical Bulletin database, access September 2023, and[2,5,6,8,12,21–35]. The CR clan meteorite bulk O isotopic compositions plot to the left of the carbonaceous chondrite anhydrous mineral lines (CCAM). The Flensburg data plot around CCAM overlapping marginally with the CR clan data, while TL, CI, and CM plot to the right of CCAM. All groups remain below the terrestrial fractionation line (TFL), except CI chondrites that plot slightly above it. The carbonates in CR chondrites have comparable δ^{18}O values to Flensburg, scatter to both higher and lower values than CI, and to lower values than TL. The CR carbonate data plot around TFL, similar to CI, but with a stronger scatter up and downwards. However, Flensburg carbonates plot far below the TFL, while the CM scatter encompasses every group. For the Δ^{17}O-ε^{54}Cr systematics, average values for groups or grouplets are plotted. The data are from[10,11,36–38]. Flensburg and CR clan members have C-like nucleosynthetic anomalies. The CR clan meteorites plot closely to each other and to CH chondrites. They all are within the same region of the C meteorite composition. The similar O isotopic composition and nucleosynthetic anomalies of CR clan meteorites reflect a similar accreting material within a relatively confined region further out in the protoplanetary disk, and, more broadly, a similar material with other C meteorites.

such that the entire cluster is assumed to originate from one depth. Thermo-chronological data points are associated with each cluster. Within a single cluster, multiple data points (if derived from one and the same meteorite) and multiple meteorites (if they are considered to be paired) can correspond to one depth. Then, for each of the clusters and for each depth the quality is determined by calculating the distances of the temperature curves $T(t,d)$ to each of the data points.

We denote the measured closure time and the closure temperature of one of the radioactive decay systems (enumerated with index i) as t_i^c and T_i^c, respectively. The temperature curve $T(t,d)$ has in general a shape with an increase on a time scale of < 1 Ma to several tens of million years until the maximum temperature T_{max} is achieved at a certain time t_{max}, followed by a cooling phase. To determine the quality of the model, we distinguish between two cases for each data point:

1. If the maximum temperature T_{max} is higher than the corresponding closure temperature T_i^c, the summed distance of the temperature curve to the data points is defined by

$$\delta^2(d) = \sum_i \left(\frac{(t_i^c - t(T_i^c))^2}{\sigma_{t,i}^2} + \frac{(T_i^c - T(t_i^c, d))^2}{\sigma_{T,i}^2} \right), \tag{3}$$

where $t(T_i^c)$ is the time at which the temperature curve passed the closure temperature T_i^c on its descending branch, $T(t_i^c, d)$ is the temperature achieved at the depth d at the closure time t^c, and $\sigma_{t,i}$ and $\sigma_{T,i}$ are the errors of the determination of the closure temperatures and cooling ages, respectively. In the case of Flensburg, the carbonates formed during the initial heating phase before the temperature maximum was reached and, thus, the data are fitted on the ascending branch of a temperature curve.

2. If the maximum temperature T_{max} does not surpass the closure temperature T_i^c throughout the model time, the distance is determined by

$$\delta^2(d) = \sum_i \left(\frac{(t_i^c - t_{max})^2}{\sigma_{t,i}^2} + \frac{(T_i^c - T_{max})^2}{\sigma_{T,i}^2} \right). \tag{4}$$

Metamorphic or melting temperatures appropriate for the meteorites involved can be utilized for defining penalty functions to penalize the depths at which the maximum temperature does not agree with the metamorphic constraints:

$$\mathscr{P} = \max\{T_{max} - T_u, 0\} - \min\{T_{max} - T_l, 0\}, \tag{5}$$

where T_u is the least upper and T_l is the highest lower bound on the metamorphic temperature. Penalty functions ensure that temperature curves with unrealistically high maxima that contradict meteorite metamorphic temperature ranges but still would fit the data well on their descending branches are excluded. The values of $\mathscr{P}$ are zero within the allowed temperature ranges and add a rapidly growing penalty the more the maximum T_{max} of a temperature curve deviates from this range. For each cluster j and for each depth, the value of $\mathscr{P}_j$ associated with a cluster is added to its value of $\delta_j^2(d)$: $\tilde{\delta}_j^2(d) = \delta_j^2(d) + \mathscr{P}_j$.

With $\tilde{\delta}_j^2 = \min_d \tilde{\delta}_j^2(d)$, we define the normalized quality function:

$$\chi_n = \left[\frac{1}{n} \sum_j \tilde{\delta}_j^2 \right]^{\frac{1}{2}} \tag{6}$$

by which we judge how good a given planetesimal fits our data set (here, n denotes the total number of the data points). The depths at which the minima $\tilde{\delta}_j^2$ are attained define the burial depths of different clusters j. Thus, our procedure determines the normalized fit quality from the best-fit depths for the meteorites within any planetesimal (equation (6)) and parameter ranges for best-fit parent bodies with their respective best-fit depths for the meteorites by minimizing the normalized fit quality for different combinations of R and t_0.

The data set for CR1-CR3 chondrites comprises two meteorites considered as two clusters - Renazzo ($j = 1$) and GRO 95577 ($j = 2$), with calcite and dolomite data for the former and dolomite data for the latter, i.e., $n = 3$. Thus, for either cluster, $\delta_j^2(d)$ has one or two summands corresponding to the respective radioactive decay systems. Since the GRO 95577 calcite age is considerably younger and is fitted quite well on the descending branch of temperature curves with unrealistically high T_{max}, we added $\mathscr{P}_2$ assuming $T_u = 400$ K without a constraint on T_l. This procedure results in one layer at the burial depth of Renazzo and one at the burial depth of GRP 95577.

The calcite and the dolomite ages derived for Flensburg[12] are indistinguishable from each other. Thus, we considered one data point, i.e., $n = 1$, as well as $\mathscr{P}_1$ with $T_l = 473$ K and $T_u = 673$ K.[12]

Data from Al-Mg, Mn-Cr, and Pb-Pb decay systems for the NWA 011 grouplet result in three data points (i.e., $n = 3$). The material of these meteorites clearly indicates silicate melting, but likely was not part of a magma ocean. Thus, $T_l = 1425$ K (silicate solidus) and $T_u = 1650$ K (minimum magma ocean temperature). The procedure produces three different layers defined by the burial depths of the three meteorites considered. A similar situation holds for the NWA 6704 grouplet but with two

meteorites and altogether four data points, i.e., two clusters and $n = 4$, and the same temperature constraints ($T_l = 1425$ K, $T_u = 1650$ K). The procedure produces two different layers defined by the burial depths of the two meteorites considered. The NWA 7680 chronology is represented by only a Mn-Cr age (i.e., $n = 1$). Thus, only one burial depth could be derived. Since this age has been shown in a conference abstract only, we do not model the parent body of NWA 7680.

Water-rich composition

The objects of modeling are parent bodies of CR chondrites and CR-like meteorites. The hydrogen abundances of CR chondrites correspond to an average water mass fraction of ≈ 4 wt.% (an average value derived from hydrogen mass fractions of single meteorites provided by[39]). The H abundances of Flensburg of 1.10 ± 0.03 wt.% correspond to a water content of ≈ 10 wt.%. Based on the petrographic type and the closed-system model,[40] for both CR and Flensburg it's a reasonable assumption that their present water contents are similar to the initial ones. For the highly metamorphosed and partially molten Tafassites as well as for the achondrites NWA 011, NWA 6704, and NWA 7680 we assume that the initial water content was similar to CR, but their parent bodies became depleted in water at least at the burial depth of this meteorite due to a high temperature. The initial water content can be uncertain if the present hydrogen abundance recorded by the Flensburg and CR chondrites represent lower bounds due to dehydration at elevated temperatures[41]. However, neither the CR chondrites, nor Flensburg were heated up to the dehydration temperature of hydrated silicates. Thus, the entire initial water content of the primordial materials has likely been consumed for hydrating dry silicates. The NWA 011 and NWA 6704 materials were heated beyond the melting temperature of silicates. Therefore, any role a potentially higher water content than measured in CR chondrites was negligible and overprinted by more energetic processes of melting, caused by a high abundance of ^{26}Al.

We include effects related to the presence of water, such as an initially ice-rich composition, consumption of the latent heat during the melting of ice, and presence of hydrous minerals upon aqueous alteration, and choose the material properties accordingly. The properties corresponding to an initial ice-olivine mixture that leads to either an average CR chondritic material rich in phyllosilicates upon aqueous alteration (with ≈ 25 vol.% phyllosilicates approximated with antigorite serpentine and ≈ 75 vol.% dry silicates approximated with olivine for CR and Tafassites, or ≈ 90 vol.% phyllosilicates and ≈ 10 vol.% dry silicates for Flensburg), or to compositions dominated by dry silicates (CR-related achondrites). The CR composition is derived by averaging the compositions of CR chondrites provided by[42]. The compositions assumed are reasonably representative for the respective meteorites in terms of the thermal evolution and compaction behavior. The consumption of the latent heat is considered in a temperature interval of two degrees between $T = 272$ K and $T = 274$ K in order to avoid numerical issues with too sharp a phase transition at 273 K. The latent heat contributes to the energy balance via the modification of the heat capacity with a Stefan number weighted with the above ice mass fraction (e.g.,[43]). The entire amount of free water obtained from the melting of ice is assumed to react quasi-instantaneously with a fraction of dry silicates to produce the phyllosilicates, in agreement with the closed-system model.[40]

While it has been suggested that water flow might have played a role for C iron meteorite parent bodies,[44] this should not be the case for the CR clan objects. Both the CR chondrites and Flensburg were not heated above the dehydration temperature of hydrated silicates and their current composition should reflect their initial water fraction. Since NWA 011 and NWA 6704 formed from the same material as CR chondrites, their initial water content should have been about the same. Since they were heated beyond the melting temperature of silicates, any role water played in their early evolution was overprinted by more energetic processes of melting, caused by a high abundance of ^{26}Al. If the initial water fraction for the NWA 011 and NWA 6704 was higher than a few wt.% and sufficient for water flow, e.g., > 25 vol.% (i.e., $>$ 10 wt.%), this water would be lost very early and very efficiently. In such a water-dominated planetesimal, solid particles would settle in the water-dominated interior in the Stokes flow regime, i.e., quasi-instantaneously, as soon as the water ice melts (e.g.,[45–47]), forming a rocky core and a water ocean atop of it. A core of an object accreting as early as the chronology and petrology of NWA 011 and NWA 6704 or iron meteorites indicates, would heat very efficiently, leading to the closure of pores within the core, extrusion of pore water into the ocean, boiling of the ocean, and an eventual loss of the water from the parent body. An ocean could be kept only if this object could not be heated strongly, e.g., due to a late accretion, as this is the case for Ceres.[48]

Accretion

Our assumption of a quasi-instantaneous parent body accretion is perfectly in line with the formation by gravitational collapse, with runaway accretion scenario that can be described by an exponential accretion law (e.g., Neumann et al. (2012,2020)[43,48]), and with the fast accretion inferred for the parent bodies of the H chondrites,[49–51] Erg Chech 002,[52] and NC iron meteorites.[53] For a scenario of an accretion spanning over up to several Ma (e.g., Elkins-Tanton et al. (2011)[54]), our results would approximate the time instance at which the majority of the parent body mass has been accreted. Such a gradual material accumulation would result in a non-trivial internal evolution in the central part of a planetesimal, but the bulk of the mass would accrete as cold primordial material at the time point of an quasi-instantaneous formation assumed in our models. Some small fraction of an already metamorphosed mass at the center can be neglected, in particular, since the meteorite fits are systematically obtained at small depths. In general, such a scenario would support our conclusion of early accretion in the C reservoir.

Impact heating

The parent bodies considered in our study could have experienced collisions with other planetesimals or leftover debris from the formation of planets. However, this could influence our results only for very specific circumstances. Of all impacts, only localized ones close to the meteorite material location within the parent body could be important for our fits. Of all those, only impacts that pre-date the closure times of the thermo-chronometers used could actually have influenced the chronometers and our fit procedure. They would be non-negligible only if they were energetic enough to reset one or multiple chronometers or produce substantial impact heating. However, no traces of impact heating or chronometer resetting have been reported for the meteorite groups considered here. Chronometer resetting can be, further, recognized with our fit procedure, as in the case of the Pb-Pb system for Tafassasset[5] and of the Ar-Ar system for the NC achondrite Erg Chech 002,[52] but we did not observe anything like that for the cases considered here. The meteorite-forming impacts that actually ejected the material from the parent bodies occurred later than the data points and they are irrelevant for our results.

Porosity

The evolution of the bulk pore space volume fraction, i.e., porosity ϕ is calculated for $T > 274$ K by considering creep of chondrules and of both hydrated and non-hydrated components of the matrix. It is described by time-dependent differential equations which establish the relation between the strain rate $\dot{\varepsilon}$ and the applied stress σ. For a mineralogical composition with the volume fractions v_k and the associated porosities ϕ_k, the average local strain rate is equal to the volume fraction weighted arithmetic mean of strain rates of the species

$$\dot{\varepsilon} = \sum_k v_k \dot{\varepsilon}_k \tag{7}$$

and the average local porosity ϕ is obtained from

$$\dot{\varepsilon} = \frac{\partial \log(1-\phi)}{\partial t}. \tag{8}$$

For CR and Tafassites, we considered three species denoted with $\phi_{i,j}$ that correspond to the matrix (antigorite) serpentine content ($i = m,\ j = se$), the matrix olivine content ($i = m,\ j = ol$), and the chondrules that were approximated with olivine ($i = ch,\ j = ol$). The creep laws for the single mineral phases provide the strain rates $\dot{\varepsilon}_{i,j}$. The serpentine strain rate is calculated from a Peierl's law equation for the deformation of antigorite at pressures of ≤ 200 MPa from[55]

$$\begin{aligned} \dot{\varepsilon}_{m,se} &= \frac{\partial \log(1-\phi_{se})}{\partial t} \\ &= 4 \cdot 10^{-22} \sigma^2 \exp\left(-\frac{27}{\mathscr{R}T}\left(1 - \frac{\sigma}{2.7 \cdot 10^9}\right)\right), \end{aligned} \tag{9}$$

while a diffusion creep law for olivine derived by[56] is used for the matrix olivine and chondrules

$$\begin{aligned} \dot{\varepsilon}_{i,ol} &= \frac{\partial \log(1-\phi_{ol})}{\partial t} \\ &= 1.26 \cdot 10^{-18} \sigma^{1.5} b_i^{-3} \exp\left(-\frac{356}{\mathscr{R}T}\right), \end{aligned} \tag{10}$$

where i is either ch or m. Here, the stress σ is in Pa, the grain size b_i in m, the activation energy $\mathscr{E}$ in kJ mol^{-1}, the gas constant $\mathscr{R}$ in kJ and the temperature T in K. In presence of iron or silicate melt, the right-hand side of equations (9) and (10) is multiplied with the term $\exp(\alpha \chi_{\mathrm{melt}})$[57], where $\alpha = 25$, χ_{melt} is the cumulate iron and silicate melt volume fraction calculated assuming linear melting between 1262 K and 1689 K for iron and 1440 K and 1736 K for silicates. The average local strain rate is $\dot{\varepsilon} = v_{m,se}\dot{\varepsilon}_{m,se} + v_{m,ol}\dot{\varepsilon}_{m,ol} + v_{ch,ol}\dot{\varepsilon}_{ch,ol}$.

The composition of Flensburg is dominated by phyllosilicates and its relict chondrutles that have a volume fraction of ≈ 20 vol.% do not contain dry silicates. Thus we do not consider them separately. As in[12], we use the antigorite power law

$$\begin{aligned} \dot{\varepsilon}_{se} &= \frac{\partial \log(1-\phi_{se})}{\partial t} \\ &= \exp(-86)\sigma^{3.8} \exp\left(-\frac{8900 + 3.2 \cdot 10^{-6} P}{\mathscr{R}T}\right), \end{aligned} \tag{11}$$

where P is the lithostatic pressure, for the phyllosilicates and the diffusion creep law for olivine (Eq. (10)) for dry silicates.

The compaction behavior of NWA 011, NWA 6704, and NWA 7680 that are dominated by dry silicates can be approximated with the olivine diffusion creep. Thus, we use Eq. (10) in these cases.

Supplementary Table 1. Parameters used in the models.

Variable	Symbol	Unit	Value	Ref.
Initial porosity	ϕ_0	-	0.5	[15]
Initial H_2O mass fraction	$x_{H_2O,0}$	-	(CR, NWA 011, NWA 6704) 0.04	[39]
			(Flensburg) 0.1	[12]
Chondrule olivine vol. fraction	$v_{ch,ol}$	-	(CR) 0.5	[58]
Matrix olivine vol. fraction	$v_{m,ol}$	-	(CR) 0.25	[58]
Matrix serpentine vol. fraction	$v_{m,se}$	-	(CR) 0.25	[58]
Matrix grain size	b_m	m	(CR, Flensburg) 10^{-6}<br>(NWA 011, NWA 6704) $7 \cdot 10^{-5}$	[12,58]<br>(...)
Chondrule grain size	b_{ch}	m	(CR) $7 \cdot 10^{-4}$	[58]
Ice th. conductivity	k_{ice}	W $m^{-1}K^{-1}$	$567/T$	[15]
Serpentine th. conductivity	k_{se}	W $m^{-1}K^{-1}$	$(0.404 + 2.46 \cdot 10^{-4} T)^{-1}$	[15]
Olivine th. conductivity	k_{ol}	W $m^{-1}K^{-1}$	4.3	[15]
Ice heat capacity	$c_{p,ice}$	J $kg^{-1}K^{-1}$	$185.0 + 7.037T$	[15]
Serpentine heat capacity	$c_{p,se}$	J $kg^{-1}K^{-1}$	$(0.9 - 6.3T^{-0.5} - 14600T^{-2}$<br>$+1.91 \cdot 10^6 T^{-3})10^4/4.52$	[15]
Chondrite heat capacity	$c_{p,c}$	J $kg^{-1}K^{-1}$	$800 + 0.25T - 1.5 \cdot 10^7 T^{-2}$	[15]
Grain density	ρ_g	kg m^{-3}	(CR) 3570<br>(Flensburg) 2695<br>(NWA 011, NWA 6704) 3690	see text<br>[12]<br>Vesta-like
Water latent heat	L_{H_2O}	J $kg^{-1}K^{-1}$	$3.34 \cdot 10^5$	
Metal solidus	$T_{Fe,S}$	K	1213	[20]
Metal liquidus	$T_{Fe,L}$	K	1700	[20]
Silicate solidus	$T_{Si,S}$	K	1425	[20]
Silicate liquidus	$T_{Si,L}$	K	1850	[20]
Ambient temperature	T_S	K	120	see text
Effective stress	σ	Pa	see text and[59]	
Gas constant	$\mathscr{R}$	J $mol^{-1}K^{-1}$	8.314472	
Stefan-Boltzmann constant	σ_{SB}	W $m^{-1}K^{-1}$	$5.67 \cdot 10^{-8}$	
Gravitational constant	G	$m^3kg^{-1}s^{-2}$	$6.67 \cdot 10^{-11}$	

Supplementary Table 2. Parameters used for the calculation of radiogenic energy. The element mass fractions are referenced in[15]. The element mass fractions refer to stable isotopes, the initial ratios are between unstable and stable isotopes of an element, and the decay energies are per particle. The number of atoms of the stable isotope per 1 kg of the primordial material is $f = xN_A/m_a$ with the relative mass fraction x of the stable isotope, the molar mass of the radioactive isotope m_a in kg, and the Avogadro number N_A.

Isotope	^{26}Al	^{60}Fe	^{40}K	^{232}Th	^{235}U	^{238}U
Element mass fr. x	$1.22 \cdot 10^{-2}$	$2.39 \cdot 10^{-1}$	$2.61 \cdot 10^{-4}$	$4.04 \cdot 10^{-8}$	$1.01 \cdot 10^{-8}$	$1.01 \cdot 10^{-8}$
Half-life λ [years]	$7.17 \cdot 10^{5}$	$2.62 \cdot 10^{6}$	$1.25 \cdot 10^{9}$	$1.41 \cdot 10^{10}$	$7.04 \cdot 10^{8}$	$4.47 \cdot 10^{9}$
Initial ratio Z	$5.25 \cdot 10^{-5}$	$1.15 \cdot 10^{-8}$	$1.50 \cdot 10^{-3}$	1.0	0.24	0.76
Decay energy E [J]	$4.99 \cdot 10^{-13}$	$4.34 \cdot 10^{-13}$	$1.11 \cdot 10^{-13}$	$6.47 \cdot 10^{-12}$	$7.11 \cdot 10^{-12}$	$7.61 \cdot 10^{-12}$

The grain size b can be derived from the chondrule or matrix material grain sizes. For CR and Tafassite calculations a grain size of $b_m = 1$ μm is based on the matrix grain sizes of CR chondrites, while $b_{ch} = 7 \cdot 10^{-4}$ m corresponds to their typical chondrule size[58]. For the Flensburg model we used a matrix grain size of 1 μm[12] and for the CR-related achondrite models a matrix grain size of 70 μm. An initial porosity of $\phi_0 = 0.5$ is a typical value based on the porosities of the random loose and random close packings (e.g.,[18,59]). The effective stress σ is calculated for a specific ordered packing of equally sized spheres[59]. Here, we use the simple cubic packing that has a porosity of ≈ 50 %. When ϕ is close to this value, the stress is unrealistically high relative to the applied stress (i.e., σ supersedes the lithostatic pressure P by more than four orders of magnitude). Therefore, we restrict it with a maximum of $10^2 P$.

Melting and Differentiation

The method of modelling the metal-silicate separation is relevant only for parent bodies of materials that were heated beyond the solidus temperature of Fe,Ni-FeS of 1213 K, i.e., for NWA 011 and 6704. Here, we use an established procedure[5,20,52] that calculates percolation of metal and silicate melts at low melt fractions and differentiation by Stokes flow at high melt fractions, i.e., in the magma ocean regime. Both mechanisms have been confirmed by the meteorite record (e.g., by the presence of Fe-FeS veins in primitive achondrites that evidence incipient metal melt percolation) and multiple laboratory experiments (see Taylor et al., 1993[60] or Neumann et al., 2012[43] for an overview). We calculate melt production between the respective solidus and a liquidus temperatures for metal and silicates (see Supplementary Table 1), where the silicate melt fraction as a function of temperature is calculated according to[61] and the metal melts linearly between the solidus and the liquidus. The metal-silicate separation occurs via melt percolation in a partially molten system and by considering Stokes settling of metal in a magma ocean. An initial grain diameter of $2 \cdot 10^{-6}$ m and grain growth[20] to a diameter of 10^{-3} m[62] is assumed for the computation of the percolation velocity. Local fractions of metal melt are relocated towards the center, while the partial silicate melt is relocated towards the surface, if the respective velocity computed is sufficiently high. The volume occupied previously by the melt is compensated by the matrix compaction into the respective direction. If differentiated layers form, they can be largely molten, thus, a magma ocean may form in the mantle and both core and mantle could experience transient convection periods.

Material Properties

The local bulk density ρ is derived from the grain density ρ_g by scaling it with the local volume filling factor $(1-\phi)$: $\rho = (1-\phi)\rho_g$. The grain density of the compacted material with $\phi = 0$, $\rho_g = 3570$ kg m^{-3} is an average value calculated for CR chondrites. Here, we simplified compositions of ten CR chondrites provided by[42] restricting them to major species olivine, pyroxene, calcite, magnetite, sulfides, iron-nickel metal, and serpentine with grain densities of 3480, 3300, 2710, 5150, 4610, 8000, and 2550 kg m^{-3}, respectively, then calculated ρ_g for each meteorite as a volume fraction weighted arithmetic mean, and then calculated an average value. Via a similar argumentation, a grain density of 2695 kg m^{-3} was used for Flensburg and 3690 kg m^{-3} for NWA 011 and NWA 6704.

The equations for the heat capacity and the thermal conductivity change at $T = 274$ K when the entire ice fraction is converted into liquid and is assumed to be consumed completely to produce serpentine. For the CR and Flensburg models and at $T \leq 274$ K,

$$c_p = \max(400, x_{H_2O,0} c_{p,ice} + (1 - x_{H_2O,0}) c_{p,c}), \quad (12)$$

$$k = f(\phi) k_{ice}^{v_{H_2O,0}} k_{ol}^{1-v_{H_2O,0}} \quad (13)$$

where the maximum is due to the divergence of $c_{p,c}$ at $T < 134$ K and the lower bound of 400 J kg^{-1}K^{-1} is consistent with the heat capacity of CI and CM analogues at the ambient/initial temperature of $T_S = 120$ K.[63] For $T > 274$ K

$$c_p = x_{m,se}c_{p,se} + (1 - x_{m,se})c_{p,c}, \tag{14}$$

$$k = f(\phi)k_{m,se}^{v_{m,se}}k_{ol}^{v_{m,ol}+v_{ch,ol}}, \tag{15}$$

where the volume fraction v_i and mass fraction x_i of a species i are related via the average grain density and the grain density of the species: $v_i = x_i\rho_g/\rho_{g,i}$. For the NWA 011 and NWA 6704 models, we neglect the contribution of water, accounting for the contribution of metal and silicates during and after the differentiation. Here, ρ, k, and c_p are calculated as in[20].

The function $f(\phi)$ varies between $\mathcal{O}(0.001)$ and 1 if the porosity varies between ϕ_0 and zero[64]:

$$f(\phi) = \left(\max\{1 - 2.216\phi, 0\}^4 + \exp\left(-1.2 - \phi/0.167\right)^4\right)^{1/4}. \tag{16}$$

Convection

While the peak metamorphic temperatures of typical CR chondrites and of Flensburg range far below typical melting temperatures of chondritic materials, the basaltic achondrites NWA 011 and NWA 6704 formed in high temperature environments. For all calculations, we consider cooling due to the liduid-state convection in a magma ocean if it forms. A magma ocean is a region where the temperature is high enough to produce more than 50 % melt. For an ordinary chondritic composition, this temperature is ≈ 1650 K. Above $T = 1650$ K, the thermal conductivity is substituted with the effective thermal conductivity k_{eff}, that simulates cooling by convection in a mixed iron-silicate magma ocean:

$$k_{eff} = 0.089kRa^{1/3}, \tag{17}$$

where Ra is the Rayleigh number (see, e.g.,[20]). A magma ocean can, in principle, form in a certain range of parameter value combinations with respect to parent body accretion time and size, but is not expected to occur for low-temperature best-fit parent bodies. Since percolation of metal and silicate melt is not indicated by the CR material and Flensburg, we do not consider any sort of core and mantle formation for those two models and use k_{eff} for regions that exceed 1650 K. For the models for NWA 011 and NWA 6704, we calculate k_{eff} for the part of the mantle that exceeds 1650 K and another k_{eff} for the part of the core that exceeds 1465 K (corresponding to 50% metal melt) and has a super-adiabatic heat flow at its top boundary.

Radius Change

The radius $\overline{R}(t)$ of the object considered changes with the bulk porosity $\phi_{bulk}(t)$ at the time t, obtained by integrating the local porosity over the radius r, according to

$$\overline{R}(t) = (1 - \phi_{bulk}(t))^{-1/3}R, \tag{18}$$

where R is the reference radius, i.e., the radius that would be attained if the porosity were zero. The reference radius is used for the analysis of the results since it is representative for sets of bodies with equal mass and grain density, but different porosity. The relation $\overline{R}(t) > R$ is always true and $\overline{R}(t) = R$ would be only possible for $\phi_{bulk}(t) = 0$, which never occurs in the calculations.

All equations involved are solved on the spatial radius domain ranging from the center of the planetesimal up to its surface. The spatial grid is transformed from $0 \leq r \leq R$, with the distance from the center r in m, to $0 \leq \eta \leq 1$ using the transformation $\eta := r/\overline{R}(t)$. The time and space derivatives are transformed as well and the transformed expressions are applied to all equations involved, such that features like Langrangian transport of porosity (Eq. (10),[43]) and other quantities are accounted for. While the positions of the grid points between 0 and 1 are fixed, the variable values at the grid points are updated at every time step according to the above transformations. The natural number N of grid points is chosen such that $[R/N] = 50$, such that a distance of ≈ 60 m between the grid points results for $\phi_0 = 0.5$. Non-stationary equations are discretized also with respect to the time variable t and solved using implicit finite difference method.

Age Calibrations

A variety of geochronological age dating systems can be used to constrain thermal evolution models of meteorite parent bodies, in the case of this study particularly U-Pb-Pb, ^{26}Al-^{26}Mg and ^{53}Mn-^{53}Cr (main paper, Table 1).

^{238}U/^{235}U-corrected ^{207}Pb/^{206}Pb Ages

The long-lived U-Pb-Pb chronometer provides the absolute time scale used for early solar system chronology, based on the decay of ^{238}U to ^{206} Pb and ^{235}U to ^{207}Pb. Besides precise measurements of Pb isotopes and U/Pb ratios, it is also important to check for possible heterogeneities in ^{238}U/^{235}U isotopic composition (particularly in refractory materials like CAIs), and - if necessary - provide U-corrected Pb-Pb ages (e.g.,[65,66]).

^{26}Al-^{26}Mg Ages

Of particular interest for parent body modeling is the time interval relative to CAIs ("time after CAIs"), as CAIs yield a canonical start value of the initial $^{26}Al/^{27}Al$ value of 5.23×10^{-5} (ref.[67]). For ^{26}Al-^{26}Mg ages, a time after CAIs can be directly inferred by relating their isochron ^{26}Al-^{26}Mg slope value to the canonical CAI value (i.e., without any other asumptions, using the value of the ^{26}Al half-life of 0.72 Ma only). The Al-Mg systematics are available for NWA 011, NWA 2976, NWA 4587, NWA 6704 and NWA 10132.

^{53}Mn-^{53}Cr Ages

For the ^{53}Mn-^{53}Cr isotopic system, we need a distinct reasoning of anchoring its relative time scale to the absolute time scale defined by the long-lived U-Pb-Pb system. As the solar system initial $^{53}Mn/^{55}Mn$ is not well constrained due to the lack of Mn in CAIs and uncertainty on their absolute U-corrected Pb-Pb age, the initial $^{53}Mn/^{55}Mn$ ratios of individual samples cannot be directly translated into ages relative to the beginning of the solar system (i.e., to the time of CAI formation). They must first be converted to absolute ages using a different anchor such as angrites. This is here exemplified based on carbonate ages in the meteorite Flensburg[12]. Relative to the angrite D'Orbigny with an initial $^{53}Mn/^{55}Mn = (3.54 \pm 0.18) \times 10^{-6}$ [68] and a (U isotope corrected) Pb-Pb age of 4563.37 ± 0.25[66], the initial $^{53}Mn/^{55}Mn$ ratios of the Flensburg carbonates correspond to absolute ages of 4564.6 ± 1.0 Ma (2σ error, including all uncertainties, e.g. relative ion yields (RIY), D'Orbigny age, ^{53}Mn decay constant). Relative to an absolute Pb-Pb age of 4567.94 ± 0.31 Ma for CAIs[69], the carbonates formed at 3.4 ± 1.0 Ma after CAI formation. This age would change to 2.6 ± 1.0 Ma if an alternative Pb-Pb age of 4567.16 ± 0.30 Ma for CAIs[70,71] is used. Both CAI-Pb-Pb ages are corrected for U isotope fractionation. However, the CAI used by[69] was analysed for ^{26}Al-^{26}Mg systematics yielding an initial $^{26}Al/^{27}Al$ ratio of $(4.9 \pm 0.03) \times 10^{-5}$ [72], consistent with a canonical value. By using the slightly older Pb-Pb age of CAIs, we follow here the argumentation by[58] that both the D'Orbigny and Sahara 99555 Pb–Pb ages are in better agreement with ^{26}Al-^{26}Mg and ^{182}Hf-^{182}W ages, as also discussed in[73].

Concerning Mn-Cr ages of carbonates in Table 1 of the main text[58,74,75], only those studies were considered in which carbonate standards were evaluated concerning relative sensitivity factors (see[12]).

References

1. Warren P. H. Stable-isotopic anomalies and the accretionary assemblage of the Earth and Mars: A subordinate role for carbonaceous chondrites. Earth and Planetary Science Letters 311, 93-100 (2011).
2. Clayton, R. N., Mayeda, T. K. Oxygen isotope studies of carbonaceous chondrites. Geochimica et Cosmochimica Acta 63, 2089-2104 (1999).
3. Clayton, R. N. Oxygen isotopes in meteorites. Annual Review of Earth and Planetary Sciences 21, 115-149 (1993).
4. Weisberg, M., Prinz, M., Clayton, R., Mayeda, T., Grady, M., Pillinger, C. The CR chondrite clan. Proceedings of the NIPR Symposium on Antarctic Meteorite 8, 11 (1995).
5. Ma, N., Neumann, W., Neri, A., Schwarz, W. H., Ludwig, T., Trieloff, M., Klahr, H., Bouvier, A. Early formation of primitive achondrites in an outer region of the protoplanetary disc Geochemical Perspective Letters 23, 33-37 (2022).
6. Schrader, D. L., Franchi, I. A., Connoly, H. C. Jr., Greenwood, R. C., Lauretta, D. S., Gibson, J. M. The formation and alteration of the Renazzo-like carbonaceous chondrites I: Implications of bulk-oxygen isotopic composition. Geochimica et Cosmochimica Acta 75, 308-325 (2011).
7. Weisberg, M. K., Prinz, M., Clayton, R. N., Mayeda, T. K. The CR (Renazzo-type) carbonaceous chondrite group and its implications. Geochimica et Cosmochimica Acta 57, 1567–1586 (1993).
8. Jilly-Rehak, C. E., Huss, G. R., Nagashima, K., Schrader, D. L. Low-temperature aqueous alteration on the CR chondrite parent body: implications from in situ oxygen-isotope analyses. Geochimica et Consmochimica Acta 222, 230-252 (2018).
9. Weiss, B. P., Elkins-Tanton, L. T. Differentiated planetesimals and the parent bodies of chondrites. Annual Review of Earth and Planetary Sciences 41, 529-260 (2013).
10. Sanborn, M. W., Wimpenny, J., Williams, C. D., Yamakawa, A., Amelin, Y., Irving, A. J., Yin, Q.-Z. Carbonaceous achondrites Northwest Africa 6704/6693: Milestones for early Solar System chronology and genealogy. Geochimica et Cosmochimica Acta 245, 577-596 (2019).
11. Huyskens, M. H., Sanborn, M. E., Yin, Q.-Z., Amelin, Y., Koefoed, P. Chronology of carbonaceous achondrites from the outer solar system. 50th Lunar and Planetary Science Conference 2019, abstract #2736 (2019).
12. Bischoff, A., et al. The old, unique C1 chondrite Flensburg – Insight into the first processes of aqueous alteration, brecciation, and the diversity of water-bearing parent bodies and lithologies. Geochimica et Cosmochimica Acta 293, 142-186 (2021).

13. Neumann, W. & Kruse, A. Differentiation of Enceladus and Retention of a Porous Core. The Astrophysical Journal 882, 47 (2019).

14. Neumann, W., Jaumann, R., Castillo-Rogez, J., Raymond, C., Russell, C. T. Ceres' partial differentiation: Undifferentiated crust mixing with a water-rich mantle. Astronomy and Astrophysics 633, A117 (2020).

15. Neumann, W., Grott, M., Trieloff, M., Jaumann, R., Biele, J., Hamm, M., Kührt, E. Microporosity and parent body of the rubble-pile NEA (162173) Ryugu. Icarus https://doi.org/10.1016/j.icarus.2020.114166, (2021).

16. Hayashi, C. Structure of the Solar Nebula, Growth and Decay of Magnetic Fields and Effects of Magnetic and Turbulent Viscosities on the Nebula. Progress of Theoretical Physics Supplement 70, 35-53 (1981).

17. Van Kooten, E., Cavalcante, L, Wielandt, D., Bizzarro, M. The role of Bells in the continuous accretion between the CM and CR chondrite reservoirs. Meteoritics and Planetary Science 55, 575-590 (2020).

18. Henke, S., Gail, H.-P., Trieloff, M., Schwarz, W. H., Kleine, T. Thermal evolution and sintering of chondritic planetesimals. Astronomy and Astrophysics 537, A45 (2012).

19. Gail, H.-P., Trieloff, M. Thermal history modelling of the L chondrite parent body. Astronomy and Astrophysics 628, A77 (2019).

20. Neumann, W., Henke, S., Breuer, D., Gail, H.-P., Schwarz, W. H., Trieloff, M., Hopp, J., Spohn, T. Modeling the evolution of the parent body of acapulcoites and lodranites: A case study for partially differentiated asteroids. Icarus 311, 146-169 (2018).

21. Rowe, M. W., Clayton, R. N., Mayeda, T. K. Oxygen isotopes in separated components of CI and CM meteorites. Geochimica et Cosmochimica Acta 58, 5341-5347 (1994).

22. Tyra, M. A., Farquhar, J., Wing, B. A., Benedix, G. K., Jull, A. J. T., Jackson, T., Thiemens, M. H. Terrestrial alteration of carbonate in a suite of Antarctic CM chondrites: Evidence from oxygen and carbon isotopes. Geochimica et Cosmochimica Acta 71, 782-795 (2007).

23. Tyra, M. A., Farquhar, J., Guan, X., Leshin, L. A. An oxygen isotope dichotomy in CM2 chondritic carbonates - A SIMS approach. Geochimica et Cosmochimica Acta 77, 383-395 (2012).

24. Tyra, M. A. Using oxygen and carbon stable isotopes, 53Mn-53Cr isotope systematics, and petrology to constrain the history of carbonates and water in the CR and CM chondrite parent bodies. University of New Mexico Albuquerque, New Mexico, UNM Digital Repository, https://digitalrepository.unm.edu (2013).

25. Tyra, M., Brearley, A., Guan, Y. Episodic carbonate precipitation in the CM chondrite ALH. Geochimica et Cosmochimica Acta 175, 195-207 (2016).

26. Baker, L., Franchi, I. A., Wright, I. P., Pillinger, C. T. The Oxygen isotopic composition of water from Tagish Lake: Its relationship to low-temperature phases and to other carbonaceous chondrites. Meteoritics and Planetary Science 37, 977-985 (2002).

27. Hyde, B. C., Tait, K. T., Nicklin, I., Grogory, D. A., Ali, A., Jabeen, I., Banerjee, N. R. Northwest Africa 7680: an ungrouped achondrite with affinities to primitive achondrite groups. 76th Annual Meteoritical Society Meeting, abstract #5207 (2013).

28. Engrand, C., Gounelle, M., Zolensky, M. E. In-SITU oxygen isotopic composition of Tagish Lake: An ungrouped type 2 carbonaceous chondrite. OAI identifier: oai:casi.ntrs.nasa.gov:20110011647, http://hdl.handle.net/2060/20110011647 (2001).

29. Leshin, L. A., Farquhar, J., Guan, Y., Pizzarello, S., Jackson, T. L., Thiemens, M. H. Oxygen isotopic anatomy of Tagish Lake: Relationship to primary and secondary minerals in CI and CM chondrites. Lunar and Planetary Science XXXII, abstract nr. 1843 (2001).

30. Yurimoto, H., Krot, A. N., Choi, B.-G., Aleon, J., Kunihiro, T., Brearley, A. J. Oxygen isotopes of chondritic components. Reviews in Mineralogy and Geochemistry 68, 141-186 (2008).

31. Piralla, M., Marrocchi, Y., Verdier-Paoletti, M. J., Vacher, L. G., Villeneuve, J., Piani, L., Bekaert, D. V., Gounelle, M. Primordial water and dust of the Solar System: Insights from in-situ oxygen measurements of CI chondrites. Geochimica et Cosmochimica Acta 269, 451-464 (2020).

32. Telus, M., Alexander, C. M., Hauri, E. H., Wang, J. Calcite and dolomite formation in the CM parent body: Insight from in situ C and O isotope analyses. Geochimica et Cosmochimica Acta 260, 275-291 (2019).

33. Lindgren, P., Lee, M. R., Starkey, N. A., Franchi, I. A. Fluid evolution in CM carbonaceous chondrites tracked through the oxygen isotopic compositions of carbonates. Geochimica et Cosmochimica Acta 204, 240-251 (2017).

34. Fujiya, W., Hoppe, P., Ushikubo, T., Fukuda, K., Lindgren, P. Lee, M. R., Koike, M., Shirai, K., Sano, Y. Migration of D-type asteroids from the outer Solar System inferred from carbonate in meteorites. Nature Astronomy 3, pages 910–915 (2019).

35. Benedix, G. K., Leshin, L. A., Farquhar, J., Jackson, T., Thiemens, M. H. Carbonates in CM2 chondrites: Constraints on alteration conditions from oxygen isotopic compositions and petrographic observations. Geochimica et Cosmochimica Acta 67, 1577-1588 (2003).

36. Dauphas, N, Schauble, E. A. Mass Fractionation Laws, Mass-Independent Effects, and Isotopic Anomalies. Annual Review of Earth and Planetary Sciences, 44, 709-783 (2016).

37. Scott, E. R. D., Krot, A. N., Sanders, I. S. Isotopic Dichotomy among Meteorites and Its Bearing on the Protoplanetary Disk. The Astrophysical Journal, 854, 164 (2018).

38. Burkhardt, C., Dauphas, N., Hans, U., Bourdon, B., Kleine, T. Elemental and isotopic variability in solar system materials by mixing and processing of primordial disk reservoirs. Geochimica et Cosmochimica Acta, 261, 145-170 (2019).

39. Alexander, C. M. O'D., Howard, K. T., Bowden, R., Fogel, M. L. The classification of CM and CR chondrites using bulk H, C and N abundances and isotopic compositions Geochimica et Cosmochimica Acta, 123, 244–260 (2013).

40. Bland, P. A., Jackson, M. D., Coker, R. F., Cohen, B. A., Webber, J. B. W., Lee, M. R., Duffy, C. M., Chater, R. J., Ardakani, M. G., McPhail, D. S., McComb, D. W., Benedix, G. K. Why aqueous alteration in asteroids was isochemical: High porosity $\neq$ high permeability. Earth and Planetary Science Letters 287, 559-568 (2009).

41. Bonal, L, Gattacceca, J., Garenne, A., Eschrig, J., Rochette, P., Ruggiu, L. K. Water and heat: New constraints on the evolution of the CV chondrite parent body. Geochimica et Cosmochimica Acta 276, 363-383 (2020).

42. Howard, K. T., Alexander, C. M. O'D., Schrader, D. L., Dyl, K. A. Classification of hydrous meteorites (CR, CM and C2 ungrouped) by phyllosilicate fraction: PSD-XRD modal mineralogy and planetesimal environments. Geochimica et Cosmochimica Acta 149, 206-222 (2015).

43. Neumann, W., Breuer, D., Spohn, T. Differentiation and core formation in accreting planetesimals. Astronomy and Astrophysics 543, A141 (2012).

44. Spitzer, F, Burkhardt, C., Nimmo, F., Kleine, T. Nucleosynthetic Pt isotope anomalies and the Hf-W chronology of core formation in inner and outer solar system planetesimals. Earth and Planetary Science Letters 576, 117211 (2021).

45. Wakita, S., Sekiya, M. Thermal evolution of icy planetesimals in the solar nebula. Earth Planets Space 63, 1193-1206 (2011).

46. Malamud, U., Prialnik, D. Modeling serpentinization: Applied to the early evolution of Enceladus Icarus 225, 763-774 (2013).

47. Neumann, W., Breuer, D., Spohn, T. Differentiation of Icy Bodies by Darcy law, Stokes law, and Two-Phase Flow. Proceedings of the International Astronomical Union 11 (A29A), 261-266 (2016).

48. Neumann, W., Jaumann, R., Castillo-Rogez, J., Raymond, C. A., Russell, C. T. Ceres' partial differentiation: undifferentiated crust mixing with a water-rich mantle. Astronomy and Astrophysics 633, A117 (2020).

49. Henke, S., Gail, H.-P., Trieloff, M., Schwarz, W. H. Thermal evolution model for the H chondrite asteroid - instantaneous formation versus protracted accretion. Icarus 226, 212-228 (2013).

50. Monnereau, M., Toplis, M. J., Baratoux, D., Guignard, J. Thermal history of the H-chondrite parent body: Implications for metamorphic grade and accretionary time-scales. Geochimica et Cosmochimica Acta 119, 302-321 (2013).

51. Pedersen, S. G., Schiller, M., Connelly, J. N., Bizzarro, M. Testing accretion mechanisms of the H chondrite parent body utilizing nucleosynthetic anomalies. Meteoritics and Planetary Science 54, 1215-1227 (2019).

52. Neumann W., Luther R., Trieloff M., Reger P. M., Bouvier A. Fitting thermal evolution models to the chronological record of Erg Chech 002 and modelling the ejection conditions of the meteorite. The Planetary Science Journal 4, 196 (2023).

53. Kleine, T., Budde, G., Burkhardt, C., Kruijer, T. S., Worsham, E. A., Morbidelli, A., Nimmo, F. The Non-carbonaceous–Carbonaceous Meteorite Dichotomy. Space Science Review 216, 55 (2020).

54. Elkins-Tanton, L. T., Weiss, B. P., Zuber, M. T. Chondrites as samples of differentiated planetesimals. Earth and Planetary Science Letters 305, 1-10 (2011).

55. Amiguet, E., Reynard, B., Caracas, R., Van de Moortele, B., Hilairet, N., Wang, Y. Creep of phyllosilicates at the onset of plate tectonics. Earth and Planetary Science Letters 345-348, 142-150 (2012).

56. Schwenn, M. B. & Goetze, C. Creep of olivine during hot-pressing. Tectonophysics 48, 41-60 (1978).

57. Mei, S., Bai, W., Hiagara, T., Kohlstedt, D. L. In£uence of melt on the creep behavior of olivine-basalt aggregates under hydrous conditions. Earth and Planetary Science Letters 201, 491-507 (2002).

58. Jilly-Rehak, C. E., Huss, G. R., Nagashima, K. 53Mn-53Cr radiometric dating of secondary carbonates in CR chondrites: Timescales for parent body aqueous alteration. Geochimica et Cosmochimica Acta 201, 224-244 (2017).

59. Neumann, W., Breuer, D., Spohn, T. Modelling of compaction in planetesimals Astronomy and Astrophysics 567, A120 (2014).

60. Taylor, G. J., Keil, K., McCoy, T., Haack, H. Scott, E. R. D. Asteroid differentiation: pyroclastic volcanism to magma ocean. Meteoritics 28, 34-52 (1993).

61. McKenzie, D. and Bickle, M. J. The volume and composition of melt generated by extension of the lithosphere. Journal of Petrology 29, 625-679 (1988).

62. McGraw, A. M., Reddy, V., Izawa, M. R. M., Sanchez, J. A., Le Corre, L., Cloutis, E. A., Applin, D. M., Pearson, N. Mineralogical criteria for the parent asteroid of the "carbonaceous" achondrite NWA 6704. The Astronomical Journal 159, 107 (11pp) (2020).

63. Biele, J., Grott, M., Zolensky, M. E., Benisek, A., Dachs, E. The specific heat of regolith material. Hayabusa2 and Osiris-Rex Workshop, 04. - 09. November 2019, Tucson, USA (2019).

64. Henke, S., Gail, H.-P., Trieloff, M. Thermal evolution and sintering of chondritic planetesimals III. Modelling the heat conductivity of porous chondrite material. Astronomy and Astrophysics, 589, A41 (2016).

65. Brennecka, G. A., Weyer, S., Wadhwa, M., Janney, P. E., Zipfel, J., Anbar, A. D. 238U/235U variations in meteorites: Extant 247Cm and implications for Pb-Pb dating. Science, 327, 449-451 (2010).

66. Brennecka, G. A., Wadhwa, M. Uranium isotope compositions of the basaltic angrite meteorites and the chronological implications for the early Solar System. Proceedings of the National Academy of Sciences of the United States of America, 109, 9299-9303 (2012).

67. Jacobsen, B., Yin, Q.-Z., Moynier, F., Amelin, Y., Krot, A., Nagashima, K., Hutcheon, I., Palme, H. 26Al–26Mg and 207Pb–206Pb systematics of Allende CAIs: Canonical solar initial 26Al/27Al ratio reinstated. Earth and Planetary Science Letters, 272, 353-364 (2008).

68. McKibbin, S. J., Ireland, T. R., Amelin, Y., Holden, P. Mn-Cr dating of Fe- and Ca-rich olivine from 'quenched' and 'plutonic' angrite meteorites using Secondary Ion Mass Spectrometry. Geochimica et Cosmochimica Acta, 157, 13-27 (2015).

69. Bouvier, A., Brennecka, G. A., Wadhwa, M. Absolute Chronology of the first solids in the solar system. Workshop on Formation of the First Solids in the Solar System, abstract #9054 (2011).

70. Connelly, J. N., Bizarro, M., Krot, A. N., Nordlund, A., Wielandt, D., Ivanova, M. A. The Absolute Chronology and Thermal Processing of Solids in the Solar Protoplanetary Disk. Science 338, 651-655 (2012).

71. Amelin, Y., Kaltenbach, A., Iizuka, T., Strling, C. H., Ireland, T. R., Petaev, M., Jacobsen, S. B. U-Pb chronology of the Solar System's oldest solids with variable ^{238}U/^{235}U. Earth and Planetary Science Letters, 300, 343-350 (2010).

72. Wadhwa, M., Kita, N. T., Nakashima, D., Bullock, E. S., MAcPherson, G. J., Bouvier, A. High precision 26Al-26Mg isotope systematics for an almost pristine refractory inclusion: Implications for the absolute age of the early solar system. 45th Lunar and Planetary Science Conference, abstract #2698 (2014).

73. Kruijer, T. S., Kleine, T., Fischer-Godde, M., Burkhardt, C., Wieler, R. Nucleosynthetic W isotope anomalies and the Hf-W chronometry of Ca-Al-rich inclusions. Earth and Planetary Science Letters, 403, 317-327 (2014).

74. Fujiya, W., Sugiura, N., Hotta, H., Ichimura, K., Sano, Y. Evidence for the late formation of hydrous asteroids from young meteoritic carbonates. Nature Communications, 3, 627 (2012).

75. Fujiya, W., Sugiura, N., Sano, Y., Hiyagon, H. Mn–Cr ages of dolomites in CI chondrites and the Tagish Lake ungrouped carbonaceous chondrite. Earth and Planetary Science Letters, 362, 130-142 (2013).
